# SIRT5 Activation and Inorganic Phosphate Binding Reduce Cancer Cell Vitality by Modulating Autophagy/Mitophagy and ROS

**DOI:** 10.3390/antiox12081635

**Published:** 2023-08-18

**Authors:** Federica Barreca, Michele Aventaggiato, Laura Vitiello, Luigi Sansone, Matteo Antonio Russo, Antonello Mai, Sergio Valente, Marco Tafani

**Affiliations:** 1 Department of Experimental Medicine, Sapienza University of Rome, 00161 Rome, Italy; federica.barreca@uniroma1.it (F.B.); michele.aventaggiato@uniroma1.it (M.A.); 2 Laboratory of Flow Cytometry, IRCCS San Raffaele Roma, Via di Val Cannuta 247, 00166 Rome, Italy; laura.vitiello@sanraffaele.it; 3 MEBIC Consortium, San Raffaele University, 00166 Rome, Italy; luigi.sansone@sanraffaele.it (L.S.); matteoantoniorusso44@gmail.com (M.A.R.); 4 Cellular and Molecular Pathology, IRCCS San Raffaele Roma, Via di Val Cannuta 247, 00166 Rome, Italy; 5 Department of Drug Chemistry and Technologies, Sapienza University of Rome, 00185 Rome, Italy; antonello.mai@uniroma1.it (A.M.); sergio.valente@uniroma1.it (S.V.)

**Keywords:** autophagy, mitophagy, ROS, sirtuins, glutamine, glutaminase, hypoxia

## Abstract

Cancer cells show increased glutamine consumption. The glutaminase (GLS) enzyme controls a limiting step in glutamine catabolism. Breast tumors, especially the triple-negative subtype, have a high expression of GLS. Our recent study demonstrated that GLS activity and ammonia production are inhibited by sirtuin 5 (SIRT5). We developed MC3138, a selective SIRT5 activator. Treatment with MC3138 mimicked the deacetylation effect mediated by SIRT5 overexpression. Moreover, GLS activity was regulated by inorganic phosphate (Pi). Considering the interconnected roles of GLS, SIRT5 and Pi in cancer growth, our hypothesis is that activation of SIRT5 and reduction in Pi could represent a valid antitumoral strategy. Treating cells with MC3138 and lanthanum acetate, a Pi chelator, decreased cell viability and clonogenicity. We also observed a modulation of MAP1LC3B and ULK1 with MC3138 and lanthanum acetate. Interestingly, inhibition of the mitophagy marker BNIP3 was observed only in the presence of MC3138. Autophagy and mitophagy modulation were accompanied by an increase in cytosolic and mitochondrial reactive oxygen species (ROS). In conclusion, our results show how SIRT5 activation and/or Pi binding can represent a valid strategy to inhibit cell proliferation by reducing glutamine metabolism and mitophagy, leading to a deleterious accumulation of ROS.

## 1. Introduction

Metabolic reprogramming is recognized as an essential characteristic of tumors [1]. An example of metabolic reprogramming is represented by the Warburg effect, in which cancer cells utilize glycolysis for energy production even in the presence of oxygen and fully functional mitochondria [2,3,4]. Apart from glycolysis, glutamine addiction and reprogramming of glutamine metabolism represent two important characteristics of tumors. Glutamine is the most abundant non-essential amino acid in plasma and contributes to virtually all biosynthetic pathways in proliferating cells [5]. Most cancers are defined as “glutamine traps”, as they tend to accumulate and consume this amino acid [6]. In cancer cells, glutamine is a major source of energy to support the high rates of protein and DNA synthesis as well as serving to produce glutathione for ROS scavenging [7,8]. Glutamine also inhibits the expression of thioredoxin, a negative regulator of glucose uptake [9]. The glutamine transporter SLC1A5 (ASCT2), a member of the sodium-dependent ASC transporter family, is overexpressed in gliomas, colorectal carcinoma, hepatocellular carcinoma cells and neuroblastoma [10]. Once in the cell, glutamine develops into glutamate and ammonia by the action of the enzyme glutaminase (GLS). Glutamate is then converted to α-ketoglutarate to enter the TCA cycle [11]. Given the role of glutamine in cancer metabolism, GLS has been indicated as a possible target for anticancer therapeutic strategies. Mammalian cells possess two GLS genes: GLS1 and GLS2. In particular, GLS1 is under the control of the MYC oncogene and gives rise to glutaminase C (GAC) and renal glutaminase (KGA) splicing variants, whereas GLS2 is controlled by p53 and generates liver-type glutaminase (LGA) and glutaminase B (GLB) [12]. The different isoforms of glutaminase can meet the metabolic needs of various types of tumors: the reduced expression or enzymatic activity of GLS produces antagonistic effects on lymphoma, glioma, breast, pancreas, non-small cell lung cancers and kidney cancers [13,14]. Recent studies have demonstrated that breast tumors, especially the triple-negative subtype, have a high expression of both GLS1 and GLS2 [12,15]. Interestingly, inorganic phosphate (Pi) increases the catalytic efficiency of GAC [16,17]. Recently, several studies have shown that Pi is six times more abundant in tumor than in normal tissue [18]. Higher concentrations of Pi are required to sustain tumor metabolism, resulting in faster tumor growth. Pi concentration is higher at the site of metastases than in the primary tumor [19]. Therefore, it has been hypothesized that the accumulation of Pi sustains tumor survival and growth by increasing GAC activity [16]. Seven sirtuins have been characterized in humans [20]. Sirtuins were first identified as class III histone deacetylase (HDAC) capable of removing acetyl groups from acetylated proteins using NAD+ as a cofactor with production of nicotinamide (NAM) and acetyl ester metabolites such as 2′-O- and3′-O-acetyl-ADP ribose (2′-AADPR) [20,21]. Sirtuins in general and mitochondrial sirtuins in particular play an important role as regulators of multiple metabolic pathways, such as those of glucose, glutamine and lipids [22]. Interestingly, the three mitochondrial sirtuins, SIRT3, SIRT4 and SIRT5 regulate glutamine metabolism differently [23]. For example, glutamate dehydrogenase (GLUD1) enzyme is activated by SIRT3 [24] and inhibited by SIRT4 in pancreatic β-cells, thereby regulating insulin secretion [25]. Even if the precise mechanism and effects are still a matter of debate, SIRT5 also controls glutamine metabolism by regulating glutaminase activity [26]. We have shown that in triple-negative breast cancer cells, SIRT5 inhibition or silencing decreased the association between GLS1 and SIRT5 while increasing glutamine metabolism, ammonia production and ammonia-induced autophagy [26]. Other studies have shown that the inhibition of SIRT5, through silencing or through inhibitors, leads to a reduction in glutaminase activity in adenocarcinoma or lung cancer cells. Furthermore, there is a decrease in ammonium, which is attributable to the decrease in GLS activity [27]. Recently, we developed MC3138, a selective SIRT5 activator. Treatment of pancreatic cancer cells with MC3138 mimicked the deacetylation effect mediated by SIRT5 overexpression with decreased levels of metabolites such as glutamine and glutamate [28]. Since SIRT5 is downregulated in human and murine pancreatic ductal adenocarcinoma (PDAC), we used MC3138 in cells and organoids of pancreatic tumors, showing an inhibition of proliferation. The combination of SIRT5 activation with gemcitabine could be a therapeutic strategy against this type of cancer [28]. To confirm the profound differences among cancer types, Wang et al. found that in colorectal cancer (CRC), overexpression of SIRT5 promotes glutamine anabolic metabolism by activating GLUD1 in a deglutarylation-dependent manner, thereby increasing CRC proliferation, survival, and xenograft tumor growth. On the contrary, SIRT5 silencing, suppressed CRC cell proliferation by inducing apoptosis and cell cycle arrest [29]. Considering the important and interconnected role of glutamine, SIRT5 and inorganic phosphate (Pi) in cancer growth and progression, our hypothesis is that activation of SIRT5 and reduction in Pi could represent a valid antitumoral strategy. Our results in breast and thyroid cancer cells show that SIRT5 activation, Pi binding or SIRT5 activation plus Pi binding greatly reduces cancer cell growth by impinging on autophagy and mitophagy and increasing ROS production.

## 2. Materials and Methods

### 2.1. Cell Culture

MDA-MB-231 human breast carcinoma, CAL-62 human anaplastic thyroid cancer and BCPAP human papillary thyroid cancer cell lines were grown in RPMI 1640 medium (Merck; St. Louis, MO, USA. R0883). The MiaPaCa human pancreatic carcinoma cell line and the HaCaT human keratinocyte cell lines were grown in DMEM medium (Merck; D6546). The SH-SY5Y human neuroblastoma cell line was grown in DMEM/F12 medium (Merck, D8437). Media were supplemented with 10% fetal bovine serum (Merck; F9665), 2 mM glutamine (Merck; G7513), 100 units/mL penicillin and 0.1 mg/mL streptomycin (Merck; P0781). Cells were detached by trypsin–EDTA solution (Merck; TA049). All cell lines were maintained at 37 °C in a humidified atmosphere of 5% CO_2_ and 95% air.

### 2.2. Treatments Protocols and Reagents

The following antibodies were used in this study: acetylated lysine (Cell Signaling; Danvers, MA, USA; 9441), BECN1 (Cell Signaling, 3738), BNIP3 (Santa Cruz Biotechnology, Dallas, TX, USA; sc-56167), GAPDH (Santa Cruz Biotechnology; sc-137179), GAC (GeneTex, Irvine, CA, USA; GTX131263), HIF-1α (Cell Signaling; 14179), MAP1LC3B (Novus Biologicals, Centennial, CO, USA; NB600-1384), PRKN (Santa Cruz Biotechnology, sc-32282), SLC20A1/2 (PiT-1/2) (Santa Cruz Biotechnology, sc-101298), SLC25A3 (Santa Cruz Biotechnology, sc-376742), ULK1 (Abcam, Cambridge, UK; ab128859), VDAC1 (Santa Cruz Biotechnology, sc-390996), pULK1 (Abcam; ab156920), peroxidase-conjugated AffiniPure goat anti-rabbit IgG (H + L) (Jackson ImmunoResearch, West Grove, PA, USA; 111-035-003), and peroxidase-conjugated AffiniPure goat anti-mouse IgG (H + L) (Jackson ImmunoResearch; 115-035-062).

Lanthanum acetate (Merck; 306339) was dissolved in distilled water, filtered and then added to a final concentration of 2 mM every 24 h and 2 h before harvesting the cells.

Cobalt (III) chloride hexahydrate (Merck; C8661) was dissolved in distilled sterile water and added to a final concentration of 200 µM for 24 h or 48 h before harvesting the cells.

Bafilomycin A_1_ (Merck, B1793) was dissolved in dimethyl sulfoxide (DMSO) and added to a final concentration of 100 nM for 2 h.

### 2.3. SIRT5 Activator

Cells were treated with 50 μM of SIRT5 activator MC3138 dissolved in DMSO for 24 and/or 48 h. The synthesis, characterization and validation of MC3138 has been previously described [30]. Control cells were treated with the same concentration of DMSO.

### 2.4. Generation of GLS1-Silenced Cells

MDA-MB-231 and CAL-62 were stably transfected using a pLKO.1 vector containing an shRNA insert targeting human GLS1 (Merck; SHCLND-NM 014905). On the first day, 2 × 10^5^ cells were seeded in a 35 mm dish. The following day, the shRNA plasmid (1 μg) was transfected into cells using FuGENE^®^ transfection reagent (Promega, Milan, Italy; E2691) according to the manufacturer’s protocol. The day after, cells were transferred to a 100 mm dish. Three days later, puromycin dihydrochloride (Gibco, Monza, Italy; A11138-03) was added at a final concentration of 2 μg/mL for stably selecting silenced clones. GAC silencing was confirmed by Western blot.

### 2.5. Trypan Blue Assay

Cells were seeded in a 100 mm dish, and once at 80–90% confluence treated as described. After 24 and 48 h, cells were collected and diluted 1:5 with trypan blue. The cell suspension was applied to a hemocytometer and counted with phase contrast microscopy (Nikon EclipseTE2000U, Nikon Netherlands, Amsterdam, The Netherlands).

### 2.6. Flow Cytometry

Cells were treated as described and washed with PBS. Cells were subsequently harvested with trypsin–EDTA, washed twice with ice-cold PBS, centrifuged at 800× *g* for 5 min at 4 °C, and fixed with precooled 70% ethanol overnight at 4 °C. Samples were stained with 50 μg/mL propidium iodide (PI, P4864; Merck) in PBS for 2 h at 4 °C cover light. Samples were acquired on an LSR Fortessa X-20 flow cytometer (Becton Dickinson, Milan, Italy). Analysis of cell cycle phases and apoptotic cells (sub-G1 fraction) was performed using BS FACSDiva™ software, v8.0.2.

### 2.7. Clonogenicity Assay

Cells were seeded in a 100 mm dish, and once at 80–90% confluence treated as described. After 24 and 48 h, cells were collected, counted and 500 plated in a 100 mm dish. After about 7 days for CAL-62 and 10 days for MDA-MB-231, plates were washed with phosphate-buffered saline (PBS; Merck; 79382) and clones fixed with 4% formaldehyde solution in PBS (Merck; F8775) at rt for 15 min. After that, the dishes were washed with PBS and clones stained for 5 min with 0.5% crystal violet (Merck; C0775). Finally, plates were washed with distilled water and air-dried. After scanning each individual dish, the colonies were counted the following day.

### 2.8. Protein Extraction and Immunoblotting

Treated and untreated cells were collected and centrifuged at 3000 rpm for 5 min. After removing the supernatant, cells were lysed in 70 µL of lysis buffer containing 50 mM Tris-Cl (Merck; 93352), 250 mM sodium chloride (NaCl, Merck; S7653), 5 mM ethylenediaminetetraacetic acid (EDTA; Merck; E6758), 0.1% Triton^®^ X-100 and 0.1 mM dithiothreitol (DTT, Merck; D9163) plus 1 mM phenylmethylsulfonyl fluoride (PMSF, Merck; 93482), protease inhibitor cocktail (PI; Merck; P8340), 1 mM sodium orthovanadate (NA_3_VO_4_, Merck; S6508) and 10 mM sodium fluoride (NaF, Merck; 201154) (lysis buffer). After 30 min on ice, samples were centrifuged at 13,000 rpm for 10 min at 4 °C and the supernatants collected. Protein concentration was determined by the Bradford assay (Bio-Rad, Hercules, CA, USA; 500-0205). An equivalent quantity of proteins was boiled for 5 min, electrophoresed onto denaturating SDS-PAGE gel and transferred onto a 0.45 μm nitrocellulose membrane (Bio-Rad; 162-0115). After blocking with 5% milk, membranes were incubated with the appropriate primary antibody overnight. The next day, after three washes with 0.1% Tween^®^ 20 (Merck; P9416) in PBS (PBST) for 30 min at rt, membranes were incubated with the appropriate secondary antibody for 1 h at rt. After 3 more washes in PBST, the detection of the relevant protein was assessed by enhanced chemiluminescence (Lite Ablot^®^ TURBO, EuroClone, Milan, Italy; EMP012001). Protein content was visualized by using either the UltraCruz Autoradiography Films (Santa Cruz Biotechnology, sc-201696) or the ChemiDocTM MP Imaging System (Bio-Rad). Densitometric analysis of the bands relative to GAPDH was performed using Image J Software v1.51 (NIH, Bethesda, MD, USA).

### 2.9. Immunofluorescence

Cells (2 × 10^5^) were plated on a coverslip and after 24 h fixed for 15 min with 4% paraformaldehyde (Immunofix, Bio-Optica, Milan, Italy; 05-K01015) in PBS, washed twice in PBS and permeabilized for 10 min with 0.5% Triton X-100 (Merck; X100) in PBS. After 1 h block with 1% bovine serum albumin (BSA, Merck; A3294) at room temperature, coverslips were incubated in a humidified chamber for 2 h at room temperature with an anti-GAC antibody (1:500, Gene Tex; GTX131263) and an anti-VDAC1 antibody (1:100 Santa Cruz Biotechnology, sc-390996). Afterward, coverslips were washed with PBS 3 times (5 min/wash) and incubated for 1 h with goat anti-rabbit IgG Alexa Fluor 555 or goat anti-mouse IgG Alexa Fluor 488 fluorescent secondary antibody (1:200, Invitrogen, Carlsbad, CA, USA). Finally, samples were washed with PBS 3 times (5 min/wash) and coverslips were mounted in ProLong Diamond Antifade Mountant (Life Technologies, Thermo Fisher Scientific, Carlsbad, CA, USA) and analyzed with an LSM510 confocal microscopy (Zeiss, Oberkochen, Germany).

### 2.10. Measurements of Reactive Oxygen Species

Cells were seeded in a 35 mm dish and either left untreated or treated as described. One hour before collection, DCFH-DA (2′7′ dichlorofluorescein diacetate) (Merck; D6883) was added at a concentration of 50 µM. Afterward, the cells were collected and resuspended in PBS and fluorescence read at an excitation of 470 nm and emission of 490–495 nm using a Glomax^®^-Multi Detection System (Promega, Milan, Italy). Mitochondrial ROS were measured by incubating the cells with MitoSOX red (mitochondrial superoxide indicator, Invitrogen, Life Technologies Corporation Eugene, Oregon M36008) dissolved in DMSO at a final concentration of 5 µM for 10 min as per the manufacturer’s instructions. Subsequently, cells were collected and washed twice in HBSS/Ca/Mg (Gibco 14025-092). Fluorescence was read by a CytoFlex flow cytometer (Beckman/Coulter, Milan, Italy) and median fluorescence intensity (MFI) was considered for graphic analysis.

### 2.11. Statistical Analysis

Data are presented as the means and standard deviations determined from three or more experiments per condition. Differences between pairs of groups were analyzed by Student’s *t*-test. The level of significance was set at *p* < 0.05.

## 3. Results

### 3.1. MC3138 and Lanthanum Acetate Effect on Cancer Cell Lines

We have previously shown that SIRT5 inhibition increases glutaminase activity and ammonia-induced autophagy [26]. For this reason, we first measured glutaminase C (GAC) protein expression in different cancer cell lines as well as in HaCaT immortalized keratinocytes. Results in Supplementary Figure S1A show that all cancer cell lines examined have a higher GAC expression than noncancerous HaCaT cells. Among cancer cells, we found the highest GAC expression in triple-negative breast cancer cells MDA-MB-231 and MiaPaCa in pancreatic cancer cells, followed by thyroid anaplastic cancer cell line CAL-62 and neuroblastoma cell line SH-SY5Y, and finally by papillary thyroid cancer cell line BCPAP. In order to unravel the role of GAC in cancer cells and to evaluate the role of the SIRT5/GAC/autophagy and mitophagy axis, GAC expression was silenced by transfecting MDA-MB-231 and CAL-62 with an shRNA plasmid. Figure S1A shows a reduction in GAC expression after transfection in these two cell lines that in CAL-62 was similar to noncancerous HaCaT cells. The same reduction was observed by immunofluorescence, also showing the colocalization of GAC with the mitochondrial outer membrane protein voltage-dependent anion channel 1 (VDAC1) (Figure S1B). Importantly, reducing GAC expression also reduced the growth of both MDA-MB-231 and CAL-62 (Figure S2). Considering that glutamine metabolism regulates autophagy, used by cancer cells to increase their survival [31], and that mitochondrial glutaminase is activated by inorganic phosphate [16,17], we investigated the possibility of modulating GAC activity and autophagy by impinging on SIRT5 and inorganic phosphate. For this reason, we employed a newly synthesized and validated SIRT5 activator called MC3138 [30]. The ability of this compound to reduce lysine acetylation was compared to that of the SIRT3 activator MC2791 [30]. MC3138 decreases global lysine acetylation compared to untreated cells (Figure S3). Then, we also used lanthanum acetate, a known phosphate chelator, to chelate inorganic phosphate to further reduce GAC activity. First, we treated MDA-MB-231 and CAL-62 cells with 2 mM lanthanum acetate and MC3138 50 µM alone or in combination for 24 and 48 h. We came up with the 2 mM lanthanum acetate and MC3138 50 µM concentration after performing a dose–response analysis followed by clonogenicity and cell mortality assays on wt and GLS1-silenced MDA-MB-231 cells, in which we documented a strong reduction in both cell vitality and colony formation (Figures S4 and S5).

### 3.2. Cell Cycle and Cell Death after MC3138 and Lanthanum Acetate Treatment

**Figure 1:**
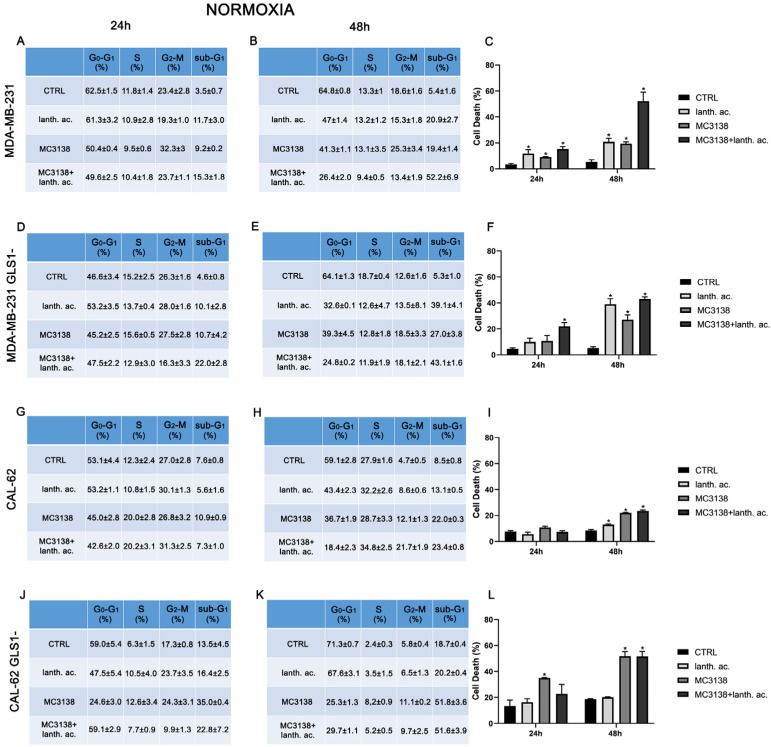
MC3138 and lanthanum acetate treatments increase cell death. (**A**,**B**) MDA-MB-231 cells were either left untreated or treated with lanthanum acetate, MC3138 or MC3138 + lanthanum acetate for 24 and 48 h. The percentage of cells in the different phases of the cell cycle was measured after propidium iodide staining and cytofluorimetric analysis. Percentages of sub-G1 cells are also reported and graphed in (**C**) showing an increase in the following treatments. (**D**,**E**) MDA-MB-231 cells silenced for GLS1 were either left untreated or treated with lanthanum acetate, MC3138 or MC3138 + lanthanum acetate for 24 and 48 h. The percentage of cells in the different phases of the cell cycle was measured after propidium iodide staining and cytofluorimetric analysis. Percentage of sub-G1 cells are also reported and graphed in (**F**) showing the increase following treatments. (**G**,**H**) CAL-62 cells were either left untreated or treated with lanthanum acetate, MC3138 or MC3138 + lanthanum acetate for 24 and 48 h. The percentage of cells in the different phases of the cell cycle was measured after propidium iodide staining and cytofluorimetric analysis. Percentage of sub-G1 cells are also reported and graphed in (**I**) showing an increase in following treatments. (**J**,**K**) CAL-62 cells silenced for GLS1 were either left untreated or treated with lanthanum acetate, MC3138 or MC3138 + lanthanum acetate for 24 and 48 h. The percentage of cells in the different phases of the cell cycle was measured after propidium iodide staining and cytofluorimetric analysis. Percentage of sub-G1 cells are also reported and graphed in (**L**) showing an increase following treatments. Experiments were repeated at least three times. Differences between pairs of groups were analyzed by Student’s *t*-test. * Significantly increased compared to control untreated cells. * *p* < 0.05. CTRL, control; lanth. ac., lanthanum acetate.

**Figure 2:**
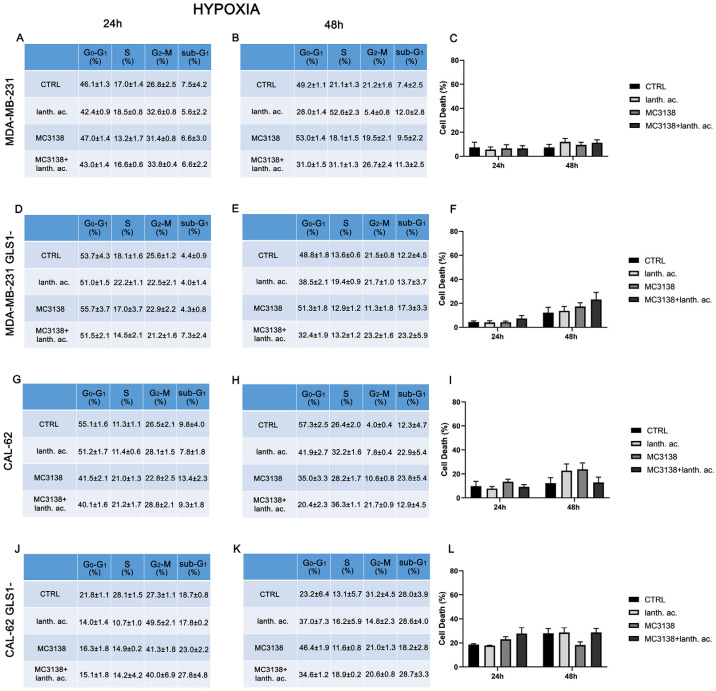
Hypoxia influences cell cycle and reduces cell death upon MC3138 and lanthanum acetate treatment. (**A**,**B**) MDA-MB-231 cells were either left untreated or treated with lanthanum acetate, MC3138 or MC3138 + lanthanum acetate under hypoxia for 24 and 48 h. The percentage of cells in the different phases of the cell cycle was measured after propidium iodide staining and cytofluorimetric analysis. Percentages of sub-G1 cells are also reported and graphed in (**C**) showing increases in the following treatments. (**D**,**E**) MDA-MB-231 cells silenced for GLS1 were either left untreated or treated with lanthanum acetate, MC3138 or MC3138 + lanthanum acetate under hypoxia for 24 and 48 h. The percentage of cells in the different phases of the cell cycle was measured after propidium iodide staining and cytofluorimetric analysis. Percentages of sub-G1 cells are also reported and graphed in (**F**) showing the increase in following treatments. (**G**,**H**) CAL-62 cells were either left untreated or treated with lanthanum acetate, MC3138 or MC3138 + lanthanum acetate under hypoxia for 24 and 48 h. The percentage of cells in the different phases of the cell cycle was measured after propidium iodide staining and cytofluorimetric analysis. Percentages of sub-G1 cells are also reported and graphed in (**I**) showing the increase in following treatments. (**J**,**K**) CAL-62 cells silenced for GLS1 were either left untreated or treated with lanthanum acetate, MC3138 or MC3138 + lanthanum acetate under hypoxia for 24 and 48 h. The percentage of cells in the different phases of the cell cycle was measured after propidium iodide staining and cytofluorimetric analysis. Percentages of sub-G1 cells are also reported and graphed in (**L**) showing the increase in following treatments. Experiments were repeated at least three times. CTRL, control; lanth. ac., lanthanum acetate.

To better understand the effect of our treatments on MDA-MB-231 and CAL-62, we decided to evaluate the cell cycle and cell death by flow cytometry after PI staining. Results shown in Figure 1 and Figure 2 indicate that while the cell cycle was not significantly influenced by 24 or 48 h treatments, there was an increase in the number of dead cells upon treatments compared to the control untreated cells.

In MDA-MB-231 after 24 h of treatment, the percentage of dead cells increased from 3.5% of the control to 11.7% with lanthanum acetate and 9.2% with MC3138 and 15.3% with MC3138 plus lanthanum acetate (Figure 1A,C). At 48 h, the percentage of cell death rose from the 5.4% of the control to 20.9% with lanthanum acetate, 19.4 of MC3138 and 52.2 of MC3138 plus lanthanum acetate (Figure 1B,C). Interestingly, compared to wt MDA-MB-231 cells, GLS1 silencing reduced cell death after 24 h of lanthanum acetate or MC3138 treatment. In MDA-MB-231 GLS1- cells, we observed a significant increase in cell death only with MC3138 plus lanthanum acetate compared to the control untreated cells (22% versus 4.6%, respectively) (Figure 1D,F). However, after 48 h, the percentage of cell death significantly increased from the 5.3% of the control to 39.1% with lanthanum acetate, 27% of MC3138 to the 43.1% of MC3138 plus lanthanum acetate (Figure 1E,F). In CAL-62 cells, we observed a significant increase in cell death only after 48 h of treatment, where the percentage of sub-G1 cells increased from the 8.5% of control untreated cells to 13.1% of lanthanum acetate, 22% of MC3138 and 23.4% of MC3138 plus lanthanum acetate (Figure 1H,I). GLS1 silencing significantly increased the percentage of dead cells from 7.6% of wt CAL-62 to the 13.5% of GLS1- after 24 h and from 8.5% of wt CAL-62 to 18.7 of GLS1- after 48 h, indicating a deleterious effect of GLS1 reduction in this cancer cell line (see Figure 1G,J and Figure 1H,K). Similarly, after 48 h, the percentage of dead cells significantly increased upon the different treatments in CAL-62 GLS1- cells, rising from the 18.7% of the control to 51.8% of MC3138 to 51.6% of MC3138 plus lanthanum acetate (Figure 1K,L). The gating strategy used to evaluate the cell cycle and cell death is reported in Supplementary Figures S6–S9.

### 3.3. Cell Cycle and Cell Death after MC3138 and Lanthanum Acetate Treatment under Hypoxia

Hypoxia and increased Pi content are two characteristics of the tumor microenvironment [19]. Moreover, Pi content increases under hypoxia [32] and hypoxia is involved in the increase in mitochondrial Pi by reducing the F1F0 ATPase activity and the Pi consumption to produce ATP [16]. Given these assumptions, we evaluated cell cycle and viability under hypoxic conditions. MDA-MB-231 and CAL-62 cells were treated with 200 µM cobalt chloride (CoCl_2_) for 24 h and 48 h. CoCl_2_ is known to mimic hypoxia in cell lines through the stabilization of HIF-1α [33]. CoCl_2_ prevents the dissolution and availability of oxygen in culture medium [33]. Hypoxic cancer cells have been shown to slowly progress to the cell cycle, accumulating in the G2–M phase [34]. In our case, we observed an increase in cells in the G2–M phase only in CAL-62 GLS1- cells (see Figure 1 and Figure 2J,K).

**Figure 3:**
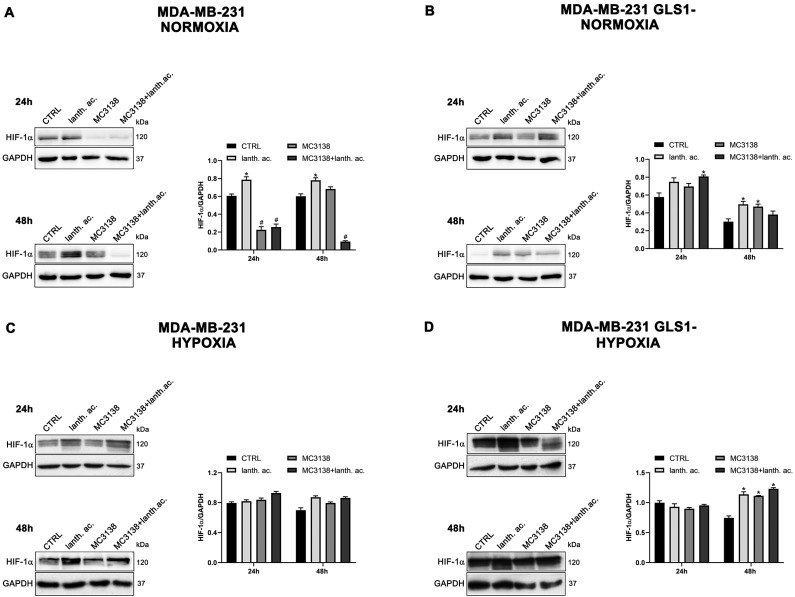
MC3138 reduces basal expression of HIF-1α in MDA-MB-231 cells. (**A**,**B**) MDA-MB-231 and MDA-MB-231 GLS1- cells were either left untreated or treated as indicated for 24 and 48 h under normoxic conditions. HIF-1α expression was determined by Western blot as described in Materials and Methods. (**C**,**D**) MDA-MB-231 and MDA-MB-231 GLS1- cells were either left untreated or treated as indicated for 24 and 48 h under hypoxic conditions. HIF-1α expression was determined by Western blot as described in Materials and Methods. GAPDH was used as loading control. HIF-1α expression was normalized with GAPDH and plotted as shown. * Significantly increased compared with untreated cells. *, *p* < 0.05. # Significantly decreased compared with control cells. #, *p* < 0.05. CTRL, control; lanth. ac., lanthanum acetate.

**Figure 4:**
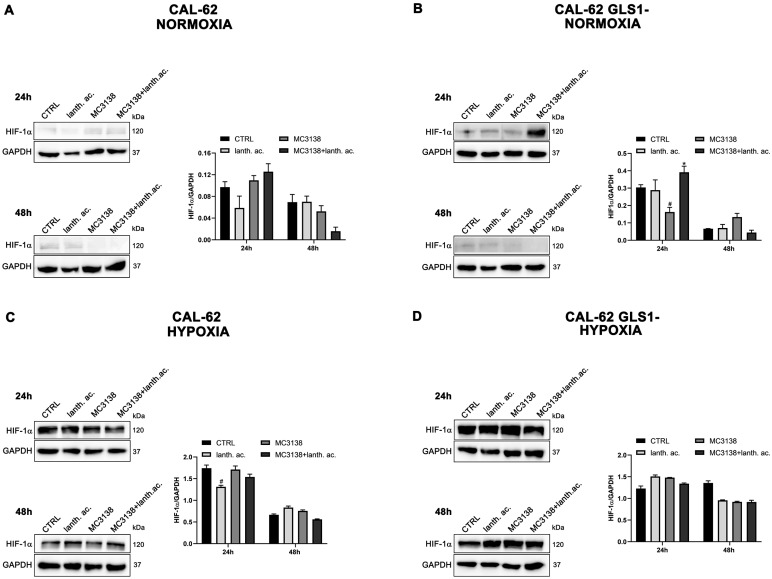
HIF-1α expression in CAL-62 cells. (**A**,**B**), CAL-62 and CAL-62 GLS1- cells were either left untreated or treated as indicated for 24 and 48 h under normoxic conditions. HIF-1α expression was determined by Western blot as described in Materials and Methods. (**C**,**D**), CAL-62 and CAL-62 GLS1- cells were either left untreated or treated as indicated for 24 and 48 h under hypoxic conditions. HIF-1α expression was determined by Western blot as described in Materials and Methods. GAPDH was used as loading control. HIF-1α expression was normalized with GAPDH and plotted as shown. * Significantly increased compared with untreated cells. *, *p* < 0.05. # Significantly decreased compared with control cells. #, *p* < 0.05. CTRL, control; lanth. ac., lanthanum acetate.

Interestingly, hypoxia increased the percentage of dead cells in the controls of both MDA-MB-231 GLS1- and CAL-62 GLS1- cells after 48 h. We measured 12.2% of dead MDA-MB-231 GLS1- control cells in hypoxia compared to 5.3% of normoxia and 28% of dead CAL-62 GLS1- control cells in hypoxia compared to 18.7% in normoxia (see Figure 1C,F,I,L). On the contrary, hypoxia reduced cell killing following treatment with lanthanum acetate, MC3138 or MC3138 plus lanthanum acetate (Figure 2). Hypoxia induction was confirmed by measuring HIF-1α expression in MDA-MB-231 and CAL-62 wt and GLS1-silenced cells. The basal expression of HIF-1α was higher in MDA-MB-231 than in CAL-62 cells (Figure 3 and Figure 4).

However, such a difference was no longer visible after CoCl_2_ treatment that resulted in HIF-1α stabilization (Figure 3 and Figure 4). Interestingly, MC3138 reduced HIF-1α basal expression in MDA-MB-231 wt cells, but not in GLS1-silenced cells (Figure 3). No significant effect was observed in CAL-62 cells, where, however, HIF-1α expression was very low (Figure 4).

### 3.4. MC3138 and Lanthanum Acetate Treatment Reduces Colony Formation

**Figure 5:**
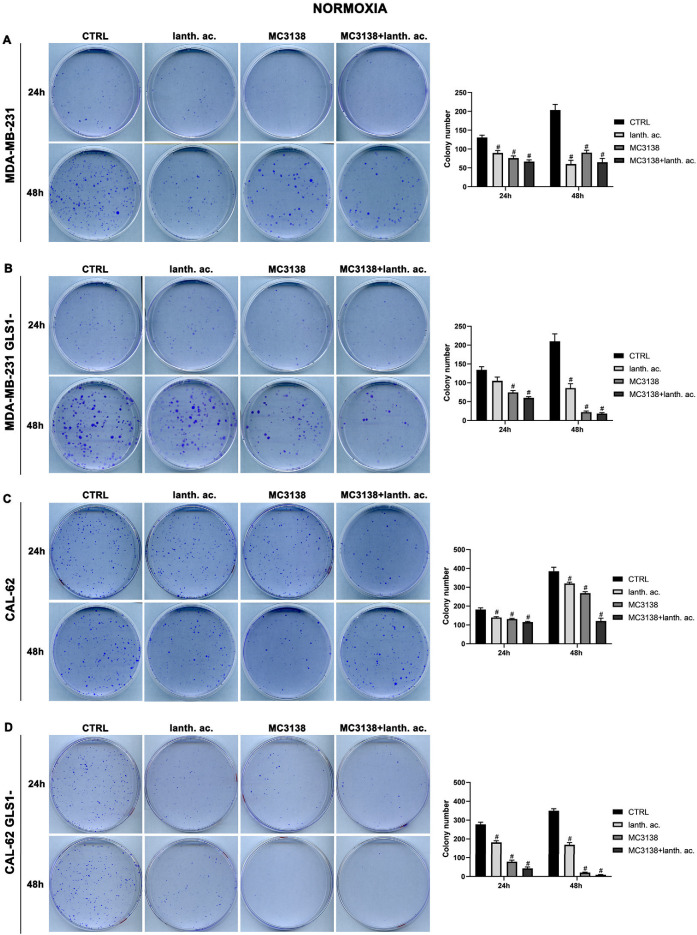
MC3138 and lanthanum acetate treatments prevent colony formation. (**A**) MDA-MB-231 cells were either left untreated or treated with lanthanum acetate, MC3138 or MC3138 + lanthanum acetate for 24 and 48 h. After 10 days, colony formation was obtained as described in Materials and Methods in 100 mm dishes and images taken. The number of colonies was counted and reported in the graph on the right side. (**B**) MDA-MB-231 GLS1- cells were either left untreated or treated with lanthanum acetate, MC3138 or MC3138 + lanthanum acetate for 24 and 48 h. After 10 days, colony formation was obtained as described in Materials and Methods in 100 mm dishes and images taken. The number of colonies was counted and reported in the graph on the right side. (**C**) CAL-62 cells were either left untreated or treated with lanthanum acetate, MC3138 or MC3138 + lanthanum acetate for 24 and 48 h. After 7 days, colony formation was obtained as described in Materials and Methods in 100 mm dishes and images taken. The number of colonies was counted and reported in the graph on the right side. (**D**) CAL-62 GLS1- cells were either left untreated or treated with lanthanum acetate, MC3138 or MC3138 + lanthanum acetate for 24 and 48 h. After 7 days, colony formation was obtained as described in Materials and Methods in 100 mm dishes and images taken. The number of colonies was counted and reported in the graph on the right side. Experiments were repeated three times. Differences between pairs of groups were analyzed by Student’s *t*-test. # Significantly decreased compared with untreated cells. #, *p* < 0.05. CTRL, control; lanth. ac., lanthanum acetate.

To determine if our treatments could affect colony formation, we performed a series of clonogenicity assays. Our results show that 24 h treatment of wt MDA-MB-231 reduced the average number of colonies from 130 in the wt, to 90 in lanthanum acetate, 76 in MC3138 and 66 in MC3138 plus lanthanum acetate (Figure 5A).

A significant reduction in the number of colonies was obtained in MDA-MB-231 GLS1- cells after 24 h treatment (Figure 5B). In CAL-62 wt cells, the number of colonies decreased significantly upon treatment with lanthanum acetate, MC3138 or MC3138 plus lanthanum acetate (Figure 5C). In CAL-62 GLS1-, the number of colonies was considerably reduced with lanthanum acetate, MC3138 and MC3138 plus lanthanum acetate (Figure 5D). The decrease in the number of colonies seen after 24 h of treatment was more defined after 48 h in MDA-MB-231, the average number of colonies decreased from 203 in the control, to 60 with lanthanum acetate, 90 with MC3138 and 65 with MC3138 plus lanthanum acetate (Figure 5A). In MDA-MB-231 GLS1-, we observed a marked decrease in the number of colonies after lanthanum acetate, MC3138 and MC3138 plus lanthanum acetate treatments (Figure 5B). For CAL-62 cells, there was an average of 385 colonies in wt that were reduced to 320 with lanthanum acetate, 268 with MC3138 and to 121 by MC3138 plus lanthanum acetate (Figure 5C). Interestingly, in CAL-62 GLS1-, there was a notable reduction in the number of colonies. Treatments reduced the average number of colonies from 350 in the control to 169 with lanthanum acetate, 20 with MC3138 to 9 with the combined treatment of MC3138 plus lanthanum acetate (Figure 5D). Overall, our results demonstrated a cytostatic effect of both single and combined MC3138 and lanthanum acetate treatment that was amplified by GLS1 silencing.

### 3.5. Colony Formation after MC3138 and Lanthanum Acetate Treatment under Hypoxia

**Figure 6:**
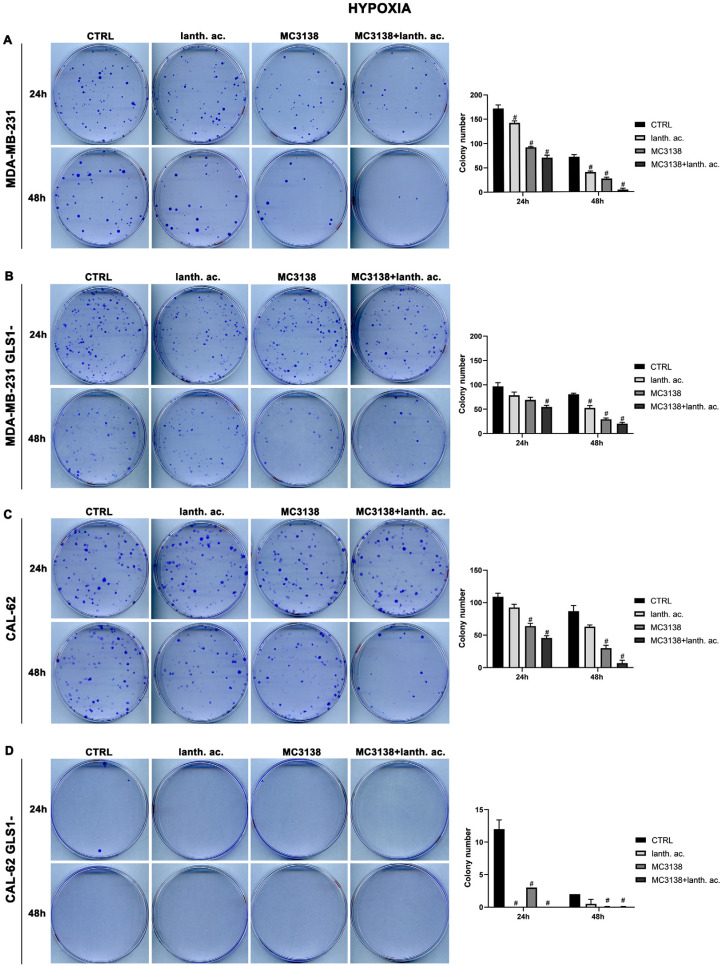
MC3138 and lanthanum acetate treatments prevent colony formation under hypoxia. (**A**) MDA-MB-231 cells were either left untreated or treated with lanthanum acetate, MC3138 or MC3138 + lanthanum acetate under hypoxia for 24 and 48 h. After 10 days, colony formation was obtained as described in Materials and Methods in 100 mm dishes and images taken. The number of colonies was counted and reported in the graph on the right side. (**B**) MDA-MB-231 GLS1- cells were either left untreated or treated with lanthanum acetate, MC3138 or MC3138 + lanthanum acetate under hypoxia for 24 and 48 h. After 10 days, colony formation was obtained as described in Materials and Methods in 100 mm dishes and images taken. The number of colonies was counted and reported in the graph on the right side. (**C**) CAL-62 cells were either left untreated or treated with lanthanum acetate, MC3138 or MC3138 + lanthanum acetate under hypoxia for 24 and 48 h. After 7 days, colony formation was obtained as described in Materials and Methods in 100 mm dishes and images taken. The number of colonies was counted and reported in the graph on the right side. (**D**) CAL-62 GLS1- cells were either left untreated or treated with lanthanum acetate, MC3138 or MC3138 + lanthanum acetate under hypoxia for 24 and 48 h. After 7 days, colony formation was obtained as described in Materials and Methods in 100 mm dishes and images taken. The number of colonies was counted and reported in the graph on the right side. Experiments were repeated three times. Differences between pairs of groups were analyzed by Student’s *t*-test. # Significantly decreased compared with untreated cells. #, *p* < 0.05. CTRL, control; lanth. ac., lanthanum acetate.

We next evaluated colony formation under hypoxia. Twenty-four hours of hypoxia did not have a relevant effect on the number of colonies in wt or GLS1- MDA-MB-231 cells when compared to normoxia (Figure 5A,B vs. Figure 6A,B).

However, we noticed an increase in colony size post-hypoxia treatment. Also under hypoxia, treatments with lanthanum acetate, MC3138 and MC3138 plus lanthanum acetate reduced the number of colonies (Figure 6A,B). In the case of wt CAL-62, there was a decrease in the number of colonies and an increase in their size following 24 h of hypoxia compared to normoxia. The average number of 181 colonies in normoxia was reduced to 109 in hypoxia (Figure 5C vs. Figure 6C). Treatments with lanthanum acetate, MC3138 and MC3138 plus lanthanum acetate reduced the average number of colonies to 92, 64 and 45, respectively (Figure 6C). Such an effect was more evident in CAL-62 GLS1-, in which the average number of colonies dropped to 12 in the control, 0 with lanthanum acetate, 3 with MC3138 and 0 with MC3138 plus lanthanum acetate (Figure 6D). As expected, a greater reduction in the average number of colonies and increase in size was observed after 48 h of hypoxia in both cell lines and GLS1- clones. In MDA-MB-231 wt, the average number of colonies was 72 in the control, 40 with lanthanum acetate, 27 with MC3138 and 4 with MC3138 plus lanthanum acetate (Figure 6A). In MDA-MB-231 GLS1-, we counted an average of 80 colonies in the control, 52 after treatment with lanthanum acetate, 29 after MC3138 and 20 with MC3138 plus lanthanum acetate (Figure 6B). A hypoxic effect was clear also in CAL-62, in which we counted 87 colonies in the control, 63 with lanthanum acetate, 30 with MC3138, and 7 with MC3138 plus lanthanum acetate (Figure 6C). Finally, the strongest effect was observed in CAL-62 GLS1-, in which there was an average of three colonies in the control, one with lanthanum acetate and zero with both MC3138 and MC3138 plus lanthanum acetate (Figure 6D). These results on GLS1-silenced clones demonstrate once again the important role of GLS1 in cancer cells and the fact that this role becomes even more important under low oxygen tension.

### 3.6. MC3138 Reduces the Expression of Phosphate Transporters

**Figure 7:**
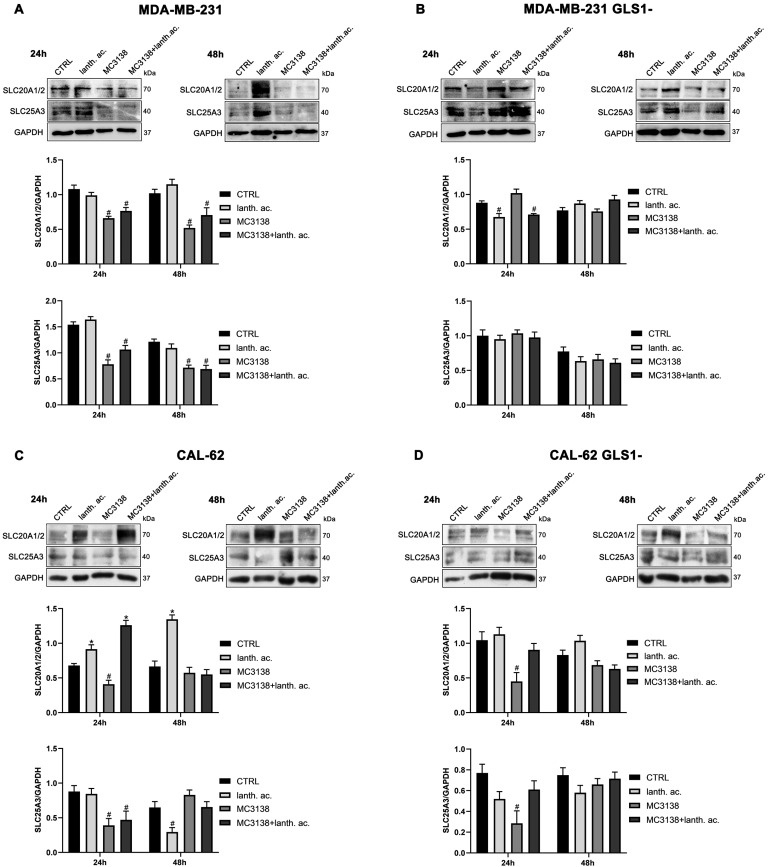
MC3138 reduces the expression of phosphate transporters. (**A**) MDA-MB-231 cells were treated as indicated in the figure. Expression levels of plasma membrane (SLC20A1 and SLC20A2 and mitochondrial (SLC25A3)) Pi transporters were determined by Western blot. Densitometric analysis of the gels was performed as described in Materials and Methods and results graphed below the blots. (**B**) MDA-MB-231 GLS1- cells were treated as indicated in the figure. Expression levels of plasma membrane (SLC20A1 and SLC20A2 and mitochondrial (SLC25A3)) Pi transporters were determined by Western blot. Densitometric analysis of the gels was performed as described in Materials and Methods and results graphed below the blots. (**C**) CAL-62 cells were treated as indicated in the figure. Expression levels of plasma membrane (SLC20A1 and SLC20A2 and mitochondrial (SLC25A3)) Pi transporters were determined by Western blot. Densitometric analysis of the gels was performed as described in Materials and Methods and results graphed below the blots. (**D**) CAL-62 GLS1- cells were treated as indicated in the figure. Expression levels of plasma membrane (SLC20A1 and SLC20A2 and mitochondrial (SLC25A3)) Pi transporters were determined by Western blot. Densitometric analysis of the gels was performed as described in Materials and Methods and results graphed below the blots. Data are representative of three separate experiments with GAPDH used as a loading control. Differences between pairs of groups were analyzed by Student’s *t*-test. * Significantly increased compared with untreated cells. *, *p* < 0.05. # Significantly decreased compared with untreated cells. #, *p* < 0.05. CTRL, control; lanth. ac., lanthanum acetate.

Since GAC requires Pi for its activity, we also determined the expression of phosphate transporters SLC20A1 and SLC20A2 localized in the plasma membrane and important for intracellular Pi homeostasis [35], as well as expression of the mitochondrial phosphate carrier SCL25A3, important for the transport of Pi in the mitochondrial matrix [36]. Our results show that SIRT5 activation through MC3138 decreased the expression of both SLC20A1, SLC20A2 and SLC25A3 in wt, but not in GLS1-silenced MDA-MB-231 cells (Figure 7A,B). In CAL-62 cells, a reduction was observed in both wt and GLS1-silenced cells (Figure 7C,D). Finally, prolonging MC3138 treatment to 48 h decreased SLC20A1, SLC20A2 and SLC25A3 expression only in wt MDA-MB-231 cells (Figure 7A).

### 3.7. Autophagy and Mitophagy Are Affected by Treatment with MC3138 and Lanthanum Acetate

**Figure 8:**
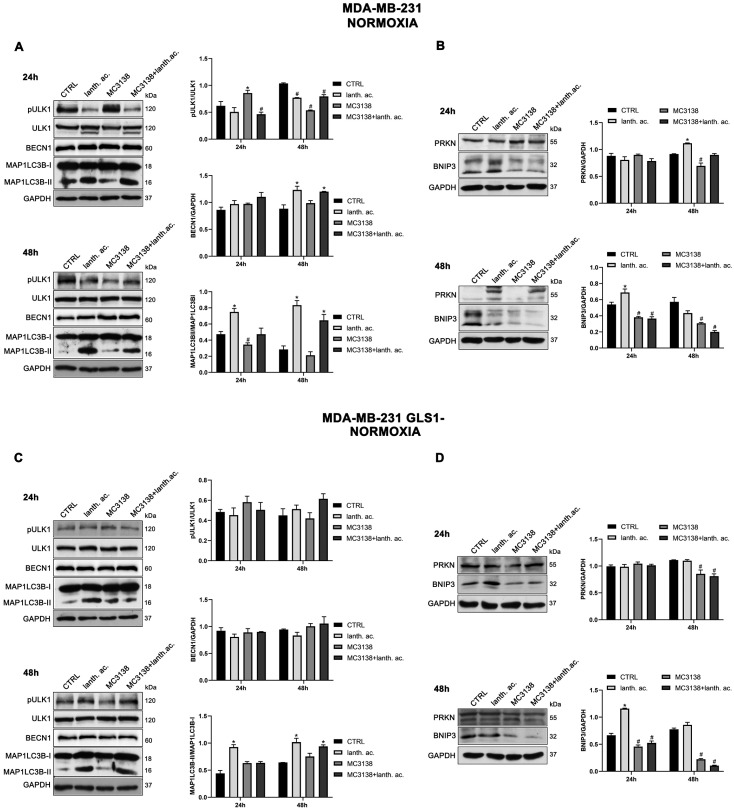
MC3138 and lanthanum acetate treatments reduce the expression of autophagy and mitophagy markers in breast cancer cells. (**A**) MDA-MB-231 cells were treated as indicated in the figure. Expression levels of autophagy proteins ULK1, phosphorylated ULK1 (pULK1), BECN1 and MAP1LC3B were determined by Western blot. Densitometric analysis of the gels was performed as described in Materials and Methods and results graphed on the right side. (**B**) MDA-MB-231 cells were treated as indicated in the figure. Expression levels of mitophagy proteins PRKN and BNIP3 were determined by Western blot. Densitometric analysis of the gels was performed as described in Materials and Methods and results graphed on the right side. (**C**) MDA-MB-231 GLS1- cells were treated as indicated in the figure. Expression levels of autophagy proteins ULK1, phosphorylated ULK (pULK1), BECN1 and MAP1LC3B were determined by Western blot. Densitometric analysis of the gels was performed as described in Materials and Methods and results graphed on the right side. (**D**) MDA-MB-231 GLS1- cells were treated as indicated in the figure. Expression levels of mitophagy proteins PRKN and BNIP3 were determined by Western blot. Densitometric analysis of the gels was performed as described in Materials and Methods and results graphed on the right side. Data are representative of three separate experiments with GAPDH used as a loading control. Differences between pairs of groups were analyzed by Student’s *t*-test. * Significantly increased compared with untreated cells. *, *p* < 0.05. # Significantly decreased compared with untreated cells. #, *p* < 0.05. CTRL, control; lanth. ac., lanthanum acetate.

**Figure 9:**
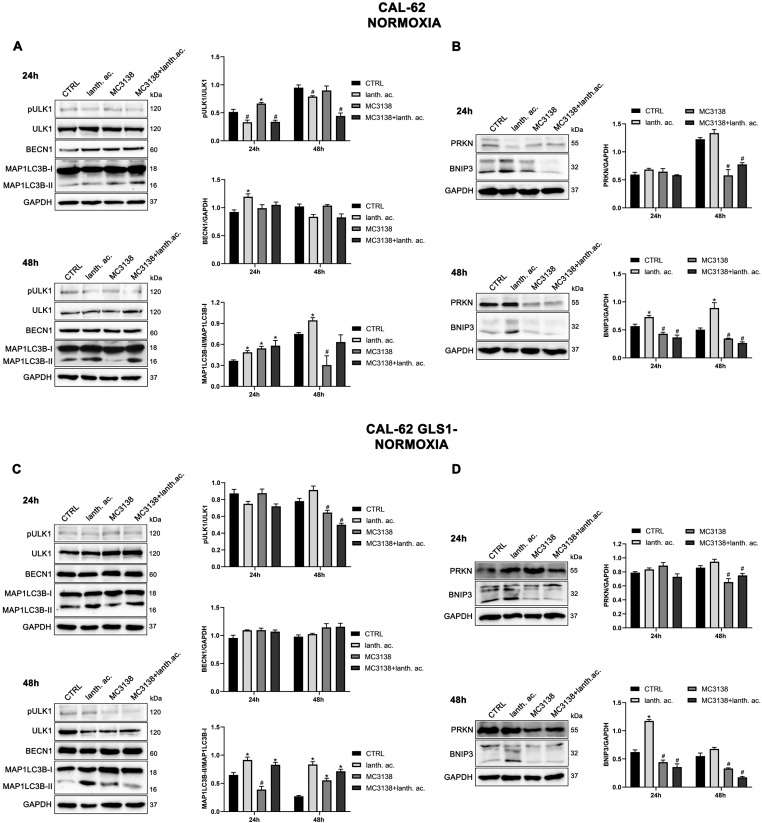
MC3138 and lanthanum acetate treatments reduce the expression of autophagy and mitophagy markers in thyroid cancer cells. (**A**) CAL-62 cells were treated as indicated in the figure. Expression levels of autophagy proteins ULK1, phosphorylated ULK1 (pULK1), BECN1 and MAP1LC3B were determined by Western blot. Densitometric analysis of the gels was performed as described in Materials and Methods and results graphed on the right side. (**B**) CAL-62 cells were treated as indicated in the figure. Expression levels of mitophagy proteins PRKN and BNIP3 were determined by Western blot. Densitometric analysis of the gels was performed as described in Materials and Methods and results graphed on the right side. (**C**) CAL-62 GLS1- cells were treated as indicated in the figure. Expression levels of autophagy proteins ULK1, phosphorylated ULK1 (pULK1), BECN1 and MAP1LC3B were determined by Western blot. Densitometric analysis of the gels was performed as described in Materials and Methods and results graphed on the right side. (**D**) CAL-62 GLS1- cells were treated as indicated in the figure. Expression levels of mitophagy proteins PRKN and BNIP3 were determined by Western blot. Densitometric analysis of the gels was performed as described in Materials and Methods and results graphed on the right side. Data are representative of three separate experiments with GAPDH used as a loading control. Differences between pairs of groups were analyzed by Student’s *t*-test. * Significantly increased compared with untreated cells. *, *p* < 0.05. # Significantly decreased compared with untreated cells. #, *p* < 0.05. CTRL, control; lanth. ac., lanthanum acetate.

SIRT5 has been associated with autophagy and mitophagy through the regulation of glutamine metabolism [37]. There are few data on the effect of inorganic phosphate on autophagy reporting that a deficiency in Pi leads to an increase in autophagy [38]. Overall, our results seem to confirm these data. Treatment of MDA-MB-231 and CAL-62 wt and GLS1- cells with lanthanum acetate for 24 h increases the autophagy marker MAP1LC3B-II (Figure 8A,C and Figure 9A,C).

In the case of MC3138, we observed a decrease in MAP1LC3B-II level in wt MDA-MB-231 and in GLS1-silenced CAL-62 cells, but not in GLS1-silenced MDA-MB-231 or CAL-62 wt cells (Figure 8A,C and Figure 9A,C). The combined treatment of MC3138 and lanthanum acetate confirmed the MAP1LC3B-II increase in the two cell lines and clones examined (Figure 8A,C and Figure 9A,C). The autophagy trend evolved after 48 h treatment. lanthanum acetate increased MAP1LC3B-II expression (Figure 8A,C and Figure 9A,C). However, the documented accumulation of MAP1LC3B-II was not due to an increase in the autophagy membranes formation, but to a block of the autophagic flux. Similarly, the decrease in MAP1LC3B-II observed with MC3138 was not due to the inhibition of autophagy membrane formation, but to an increase in the autophagic flux [39]. To unravel this, we inhibited the autophagic process using bafilomycin A_1_, which blocks both autophagosome–lysosome fusion and lysosome acidification [39]. Our results are shown in Supplementary Figures S10 and S11. In MDA-MB-231 wt control cells grown for 24 or 48 h and then treated with bafilomycin A_1_ for 2 h, we observed an accumulation of MAP1LC3B-II (Figure S10, upper panel). Bafilomycin A_1_ plus lanthanum acetate further increased MAP1LC3B-II compared to lanthanum acetate or bafilomycin A_1_ alone, suggesting that MAP1LC3B-II accumulation seen with lanthanum acetate is due to an increase in autophagy membrane formation and not to an inhibition of autophagic flux (Figure S10, upper panel). On the contrary, bafilomycin A_1_ plus MC3138 did not increase MAP1LC3B-II compared to MC3138 or bafilomycin A_1_ alone, suggesting that the decrease in MAP1LC3B-II seen with MC3138 is due to the inhibition of autophagy membrane formation and not to an increase in autophagic flux (Figure S10, upper panel). Results with lanthanum acetate plus MC3138 in the presence of bafilomycin A_1_ were similar to MC3138 alone, with no increase in MAP1LC3B-II compared to bafilomycin A_1_ alone (Figure S10, upper panel). Interestingly, in MDA-MB-231, GLS1 silencing reversed only the effect of MC3138. In MDA-MB-231 GLS1- cells, MC3138 plus bafilomycin A_1_ increased MAP1LC3B-II compared to MC3138 or bafilomycin A_1_ alone, suggesting a lack of inhibition by MC3138 of the autophagic process in the absence of GLS1 (Figure S10, lower panel). In CAL-62 wt cells, we observed the same results reported for MDA-MB-231 cells after 24 h of treatment. At this time point, the bafilomycin A_1_ treatment revealed a block of the autophagic membrane formation only in the presence of MC3138 (Figure S11, upper panel). Such an effect of MC3138 was maintained after 48 h (Figure S11). In Cal-62 cells, GLS1 silencing reversed the accumulation of MAP1LC3B in the presence of MC3138 plus bafilomycin A_1_ compared to the wt (Figure S11). in CAL-62 GLS1- cells, MAP1LC3B increased after MC3138 plus bafilomycin A_1_ treatment compared to the same treatment in the wt CAL-62 cells (Figure S11, upper and lower panels).

Minor differences in protein expression were observed for BECN1 (Figure 8A,C and Figure 9A,C). In MDA-MB-231 and CAL-62 cells, we also observed an increase in ULK1 phosphorylation at Ser 757 with MC3138 and a decrease with lanthanum acetate (Figure 8A,C and Figure 9A,C). No differences were observed in GLS1- clones (Figure 8A,C and Figure 9A,C). Since phosphorylation of Ser 757 by MTOR disrupts the interaction of ULK1 and PRKAA2 (AMPK) and inhibits autophagy [40], these data confirm what was seen with LC3 II in wt cells. Given the fact that SIRT5 is a mitochondrial sirtuin and since the metabolism of glutamine takes place within the mitochondria, we studied the effect of our treatments on the mitophagic pathway. Our results show an increase in BNIP3 after 24 h treatment with lanthanum acetate in the cell lines studied (Figure 8B,D and Figure 9B,D). On the contrary, 24 h treatment with MC3138 or MC3138 plus lanthanum acetate decreased the mitophagy marker BNIP3 (Figure 8B,D and Figure 9B,D). The autophagy and mitophagy trend evolved after 48 h treatment. Lanthanum acetate increased MAP1LC3B-II expression (Figure 8A,C and Figure 9A,C). ULK1 phosphorylation on Ser 757 was still reduced by lanthanum acetate (Figure 8A,C and Figure 9A,C). Importantly, MC3138 still decreased BNIP3 expression in wt and GLS1- MDA-MB-231 and CAL-62 cells, whereas lanthanum acetate increased BNIP3 (Figure 8B,D and Figure 9B,D). Finally, after 48 h treatment, MC3138 also decreased Parkin expression in the two cell lines and GLS1- clones studied, suggesting the need for longer treatment for an effect on this protein (Figure 8B,D and Figure 9B,D).

### 3.8. Autophagy and Mitophagy with MC3138 and Lanthanum Acetate in the Presence of Hypoxia

**Figure 10:**
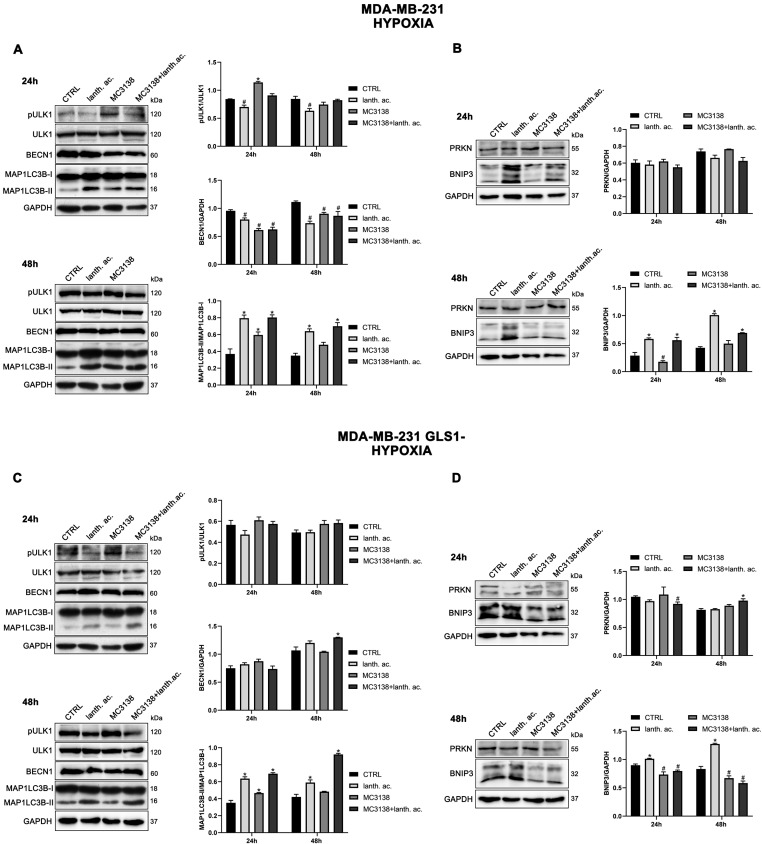
Modulation of autophagy and mitophagy markers in breast cancer cells treated with MC3138 and lanthanum acetate under hypoxia. (**A**) MDA-MB-231 cells were treated under hypoxia as indicated in the figure. Expression levels of autophagy proteins ULK1, phosphorylated ULK1 (pULK1), BECN1 and MAP1LC3B were determined by Western blot. Densitometric analysis of the gels was performed as described in Materials and Methods and results graphed on the right side. (**B**) MDA-MB-231 cells were treated under hypoxia as indicated in the figure. Expression levels of mitophagy proteins PRKN and BNIP3 were determined by Western blot. Densitometric analysis of the gels was performed as described in Materials and Methods and results graphed on the right side. (**C**) MDA-MB-231 GLS1- cells were treated under hypoxia as indicated in the figure. Expression levels of autophagy proteins ULK1, phosphorylated ULK1 (pULK1), BECN1 and MAP1LC3B were determined by Western blot. Densitometric analysis of the gels was performed as described in Materials and Methods and results graphed on the right side. (**D**) MDA-MB-231 GLS1- cells were treated under hypoxia as indicated in the figure. Expression levels of mitophagy proteins PRKN and BNIP3 were determined by Western blot. Densitometric analysis of the gels was performed as described in Materials and Methods and results graphed on the right side. Data are representative of three separate experiments with GAPDH used as a loading control. Differences between pairs of groups were analyzed by Student’s *t*-test. * Significantly increased compared with untreated cells. *, *p* < 0.05. # Significantly decreased compared with untreated cells. #, *p* < 0.05. CTRL, control; lanth. ac., lanthanum acetate.

**Figure 11:**
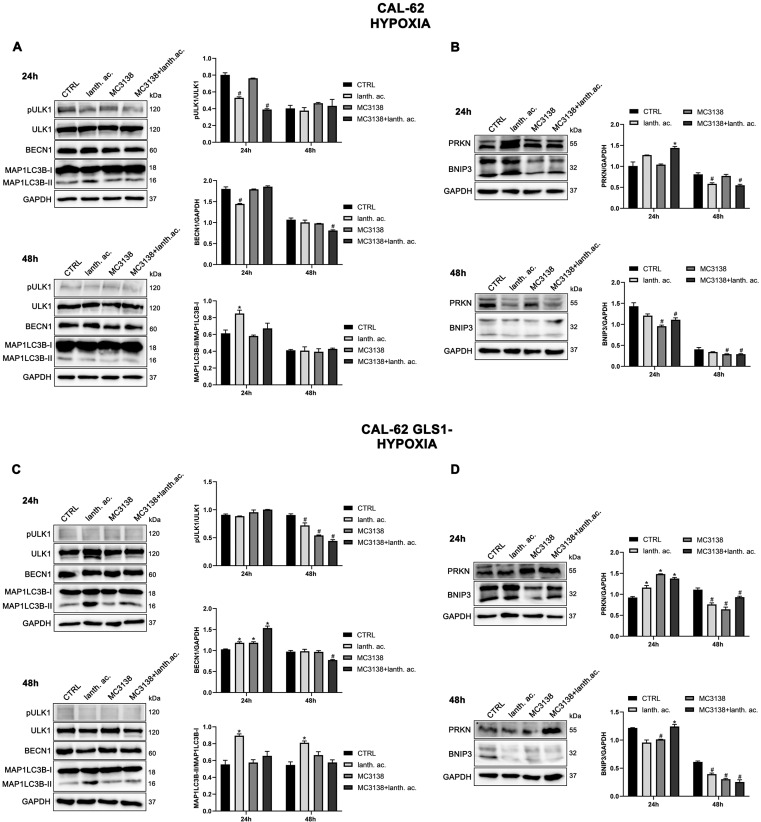
Modulation of autophagy and mitophagy markers in thyroid cancer cells treated with MC3138 and lanthanum acetate under hypoxia. (**A**) CAL-62 cells were treated under hypoxia as indicated in the figure. Expression levels of autophagy proteins ULK1, phosphorylated ULK1 (pULK1), BECN1 and MAP1LC3B were determined by Western blot. Densitometric analysis of the gels was performed as described in Materials and Methods and results graphed on the right side. (**B**) CAL-62 cells were treated under hypoxia as indicated in the figure. Expression levels of mitophagy proteins PRKN and BNIP3 were determined by Western blot. Densitometric analysis of the gels was performed as described in Materials and Methods and results graphed on the right side. (**C**) CAL-62 GLS1- cells were treated under hypoxia as indicated in the figure. Expression levels of autophagy proteins ULK1, phosphorylated ULK1 (pULK1), BECN1 and MAP1LC3B were determined by Western blot. Densitometric analysis of the gels was performed as described in Materials and Methods and results graphed on the right side. (**D**) CAL-62 GLS1- cells were treated under hypoxia as indicated in the figure. Expression levels of mitophagy proteins PRKN and BNIP3 were determined by Western blot. Densitometric analysis of the gels was performed as described in Materials and Methods and results graphed on the right side. Data are representative of three separate experiments with GAPDH used as a loading control. Differences between pairs of groups were analyzed by Student’s *t*-test. * Significantly increased compared with untreated cells. *, *p* < 0.05. # Significantly decreased compared with untreated cells. #, *p* < 0.05. CTRL, control; lanth. ac., lanthanum acetate.

It is known that hypoxia can increase autophagy and mitophagy in cancer cells [41]. In addition, hypoxia also increases mitochondrial Pi content [32]. After 24 h of hypoxia, we still observed an increase in the expression of MAP1LC3B-II in the presence of lanthanum acetate alone or in combination with MC3138 in the two cell lines and GLS1-silenced clones examined (Figure 10A,C and Figure 11A,C).

In hypoxia, MC3138 increased MAP1LC3B-II expression in MDA-MB-231 wt and GLS1- cells (Figure 10A,C). MC3138 decreased Beclin1 expression while increasing ULK1 Ser 757 phosphorylation in MDA-MB-231 cells (Figure 10A,C). Results were clearer with the mitophagy marker BNIP3. MC3138 still decreased BNIP3 expression in all the cell lines and clones studied (Figure 10B,D and Figure 11B,D). On the contrary, lanthanum acetate increased BNIP3 expression in MDA-MB-231 cells (Figure 10B,D). No relevant changes were observed for Parkin (Figure 10B,D and Figure 11B,D). The same trend for autophagy and mitophagy after lanthanum acetate and MC3138 treatment was maintained after 48 h. Again, major effects of MC3138 treatment were on mitophagy inhibition, as documented by BNIP3 decrease (Figure 10B,D and Figure 11B,D).

### 3.9. MC3138 and Lanthanum Acetate Increase Cytosolic and Mitochondrial ROS under Normoxia and Hypoxia

**Figure 12:**
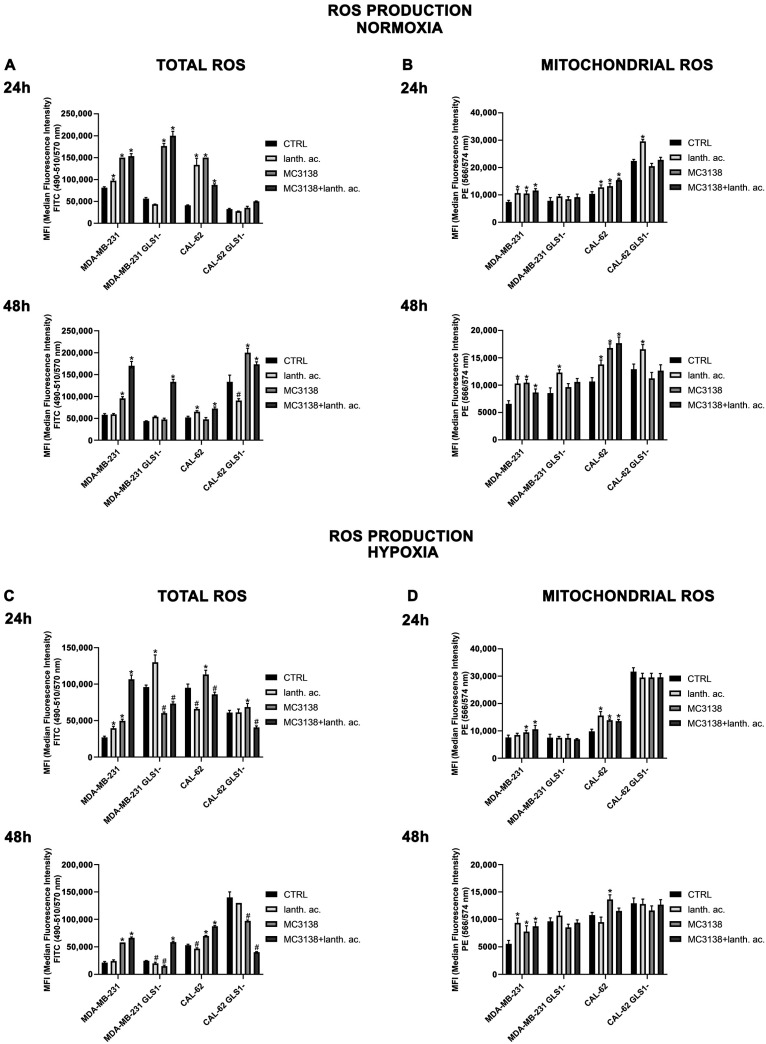
MC3138 and lanthanum acetate treatments increase cytosolic and mitochondrial ROS in cancer cells under normoxic and hypoxic conditions (**A**) MDA-MB-231, CAL-62 and GLS1- clones were either left untreated or treated with lanthanum acetate, MC3138 or MC3138 + lanthanum acetate for 24 (**upper left**) and 48 h (**lower left**). Total ROS content was measured using DCFH-DA as indicated in Materials and Methods and graphed as mean fluorescence intensity (MFI). (**B**) MDA-MB-231, CAL-62 and GLS1- clones were either left untreated or treated with lanthanum acetate, MC3138 or MC3138 + lanthanum acetate for 24 (**upper right**) and 48 h (**lower right**). Mitochondrial ROS content was measured using MitoSOX Red as indicated in Materials and Methods and graphed as mean fluorescence intensity (MFI). (**C**) MDA-MB-231, CAL-62 and GLS1- clones were either left untreated or treated with lanthanum acetate, MC3138 or MC3138 + lanthanum acetate for 24 (**upper left**) and 48 h (**lower left**) under hypoxia. Total ROS content was measured using DCFH-DA as indicated in Materials and Methods and graphed as median fluorescence intensity (MFI). (**D**) MDA-MB-231, CAL-62 and GLS1- clones were either left untreated or treated with lanthanum acetate, MC3138 or MC3138 + lanthanum acetate for 24 (**upper right**) and 48 h (**lower right**) under hypoxia. Mitochondrial ROS content was measured using MitoSOX Red as indicated in Materials and Methods and graphed as median fluorescence intensity (MFI). Data are representative of three separate experiments. Differences between pairs of groups were analyzed by Student’s *t*-test. * Significantly increased compared with untreated cells. *, *p* < 0.05. # Significantly decreased compared with untreated cells. #, *p* < 0.05. CTRL, control; lanth. ac., lanthanum acetate.

Cancer cells express high levels of antioxidant proteins to cope with elevated ROS content and to maintain homeostasis to prevent oxidative stress-induced tumor cell death [42]. The increased glutamine metabolism observed in cancer cells maintains ROS homeostasis through the accumulation of the antioxidant glutathione [43]. Therefore, activating SIRT5 and reducing Pi should result in increased ROS. Our results show that 24 h of MC3138 or MC3138 plus lanthanum acetate treatmen, significantly increased ROS levels in wt and GLS1-silenced MDA-MB-231 (Figure 12A).

Interestingly, lanthanum acetate increased ROS production in MDA-MB-231 wt but not in GLS1-silenced cells (Figure 10A). ROS levels increased also in CAL-62 wt cells (Figure 12A). However, in this cell line, GLS1 silencing did not result in a further ROS increase (Figure 12A). Interestingly, after 48 h of treatment, we observed that ROS levels decreased in MDA-MB-231 GLS1- cells compared to wt and increased in CAL-62 GLS1- cells compared to wt (Figure 12A, lower graph), indicating a different time course in ROS production between these two cell lines. However, we still observed a ROS increase in the presence of MC3138 in the two cell lines studied (Figure 12A, lower graph). Since SIRT5 is a mitochondrial sirtuin and to further investigated ROS production, we measured mitochondrial ROS accumulation with the MitoSOX Red probe. Our results show that 24 and 48 h of lanthanum acetate, MC3138 and MC3138 plus lanthanum acetate treatment increased mitochondrial ROS in both MDA-MB-231 and CAL-62 wt cells. Interestingly, such an increase was observed only with lanthanum acetate in GLS1-silenced cells (Figure 12B). Similar results were obtained in the presence of hypoxia with the difference being that the hypoxic cells showed lower ROS levels compared to normoxic after both 24 and 48 h (Figure 12C). Notwithstanding, MC3138 alone or in combination with lanthanum acetate was still able to increase ROS in the cancer cell lines examined apart from GLS1-silenced clones where the ROS decrease was due to elevated cell death (Figure 12C). In the case of mitochondrial ROS, hypoxia did not influence their content. However, compared to control, our treatments and in particular MC3138 increased mitochondrial ROS in wt MDA-MB-231 and CAL-62 cells after both 24 and 48 h (Figure 12D). It must be noted that in control untreated cells, mitochondrial ROS were higher in GLS1-silenced cells than wt cells, suggesting again the important role of glutamine metabolism in ROS scavenging (Figure 12D).

## 4. Discussion

Cancer cells take advantage of glutamine metabolism and autophagy/mitophagy activation to survive the harsh tumor microenvironment [26,31,44]. Cancer cells concentrate inorganic phosphate in the tumor microenvironment to sustain their rapid growth [19] as well as to increase the activity of mitochondrial glutaminase, the first enzyme in the glutamine metabolic pathway [16]. Recently, we have shown that mitochondrial glutaminase activity can be influenced by sirtuin 5 [26]. In this direction, our present results indicate that the use of a selective activator of sirtuin 5, called MC3138, alone or in association with a phosphate binder such as lanthanum acetate, can reduce cell viability, colony formation and mitophagy of breast and thyroid cancer cells. The use of cell lines from two different tumors evidenced a different response to the treatments. MDA-MB-231 cells show a higher increase in cell death and decrease in colony formation than CAL-62 cells (Figure 1, Figure 2, Figure 5 and Figure 6). The antitumoral effects of MC3138 and lanthanum acetate were observed after 24 h in breast cancer cells and after 48 h in thyroid cancer cells (Figure 1). In order to better understand the role of GLS1 in cancer cells as well as the connection between GLS1 and SIRT5, we generated GLS1-silenced clones of MDA-MB-231 and CAL-62 cells. Glutaminase 1 silencing significantly reduced colony formation in both cell lines (Figure 5). GLS1 silencing partially prevented the effects of SIRT5 activation. Such a partial modification is probably due to fact that SIRT5 has multiple targets in the cell in general and in the mitochondria in particular, which can mask those due to GLS1 silencing. However, we were able to observe important modifications in cell vitality, autophagy and mitophagy following GLS1 silencing. Another point is that to date, inorganic phosphate is considered a critical metabolic molecule that increases cell viability and the formation of metastases, and the literature suggests that it may be a possible predictive marker in the case of breast cancer. Inorganic phosphate is six times more concentrated in the tumor microenvironment, and for this reason, its presence could be used as a tumor marker as well as a predictive microscopic molecular biomarker for the assessment of the relative risk of malignant transformation of pretumor lesions [19]. Our results confirm the important role of inorganic phosphate for tumor survival and growth and suggest that lanthanum or other chelators could be used to reduce the presence of inorganic phosphate in the tumor. Phosphate chelation, however, raises problems related to a systemic reduction in inorganic phosphate. Recently, Qiu-Chen et al. proposed a new technique for the administration of a phosphate binder called “transarterial sevelamer embolization (TASE).” This technique not only occludes the tumor-feeding vessel, but simultaneously depletes intratumoral inorganic phosphate (Pi), thereby inducing severe necrosis as well as reducing metastasis formation and recurrence in liver cancer [45]. We also evaluated the expression of the membrane Pi transporters PiT-1/SLC20A1 and PiT-2/SLC20A2 and the mitochondrial transporter SLC25A3, important for the cellular and mitochondrial uptake of Pi. Our results show that MC3138 decreases the expression of both membrane and mitochondrial Pi transporters in MDA-MB-231 and in CAL-62 cells (Figure 7). Moreover, when treated with MC3138, wt MDA-MB-231 showed a greater reduction in the expression of PiT-1/SLC20A1, PiT-2/SLC20A2 and SLC25A3 than GLS1-silenced MDA-MB-231 after 24 h and 48 h of treatment. On the contrary, in CAL-62, wt or GLS1-silenced, such a reduction is observed only after 24 h of treatment. Our results suggest for the first time a correlation between the activity of sirtuin 5 and the expression of phosphate transporters. Interestingly, these results connect and expand upon a recent observation that in cardiomyocytes, SLC25A3 silencing increases acylation of mitochondrial proteins by decreasing SIRT5 activity and bringing activation of IDH2 [36]. Our results suggest that such an effect could work in both ways, with activation of SIRT5 causing a decrease in SLC25A3 expression. It should be taken into consideration that MDA-MB-231 has higher levels of GAC than CAL-62 (Figure S1A). The differences in term of Pi transporters and glutaminase levels between these two cancer cell lines may also reflect a different sensitivity to treatments. Overall, wt and GLS1-silenced MDA-MB-231 cells appear to be more sensitive to lanthanum acetate in terms of cell death, whereas CAL-62 cells present a greater percentage of dead cells with MC3138 treatment. Hypoxia represents another important feature of the tumor microenvironment. Hypoxia increases the demand for inorganic phosphate to sustain tumor cell growth [16,32]. Our results demonstrated a reduction in the number of colonies in both MDA-MB-231 and CAL-62 cell lines when the treatments were administered in hypoxic conditions (Figure 6), an effect particularly evident in GLS1-silenced cells. We have previously shown that by acting on glutaminase, SIRT5 also regulates ammonia-induced autophagy [23,26]. Our results confirm the involvement of autophagy following modulation of glutamine metabolism either through MC3138 or lanthanum acetate treatment in both MDA-MB-231 and CAL-62 cell lines (Figure 8 and Figure 9). By using bafilomycin A_1_, a known inhibitor of autophagosome-lysosome fusion and lysosome acidification, we demonstrated that the MAP1LC3B-II decrease observed with MC3138 was due to an inhibition of autophagy membrane formation and not to an increase in autophagic flux. Interestingly, in MDA-MB-231 and CAL-62 GLS1-silenced cells, MC3138 did not inhibit bafilomycin A_1_-induced accumulation of MAP1LC3B, suggesting that the effects of SIRT5 on autophagy may depend on this enzyme (Figures S10 and S11).

Importantly, our results indicate that the mitophagy marker BNIP3 is more affected by the treatments carried out in this study. After 48 h, both in breast and thyroid tumor cells, there is an evident reduction in the expression of BNIP3 after treatment with MC3138. This suggests that SIRT5 activation reduces mitophagy. If MC3138 treatment is coupled with lanthanum acetate, BNIP3 expression is almost undetectable, suggesting the accumulation of dysfunctional mitochondria. Sirtuins induce posttranslation modifications of autophagic and mitophagic proteins, thereby modulating such homeostatic mechanisms [37]. SIRT5 is associated with autophagy and mitophagy through the regulation of glutamine metabolism. Polletta et al., stated that SIRT5 inhibition leads to an increase in ammonia production, which in turn stimulates autophagy and mitophagy, conferring increasing benefit to cancer cells [26]. Our results confirm this finding, since by activating SIRT5 with MC3138, a relevant decrease in mitophagy is observed, which also reduces cancer cell vitality and proliferative capacity. Mitophagy activation is used by cancer cells as a protective mechanism from insults such as hypoxia. The hypoxic tumor microenvironment increases mitophagy [42]. Hypoxia activates the metastatic process supported by mitophagy [46]. We observed a decrease in the mitophagy marker BNIP3 when the cells were treated with MC3138 alone or in combination with lanthanum acetate, an effect that was more evident under hypoxia (Figure 8, Figure 9, Figure 10 and Figure 11). Cancer cells use glutamine metabolism and mitophagy to contain ROS levels by producing glutathione and removing dysfunctional mitochondria, respectively [44]. Therefore, inhibition of one or both mechanisms would result in a toxic ROS increase for cancer cells [42]. This is confirmed by our results: even with differences in terms of time (24 or 48 h) or response to hypoxia treatment, GLS1-silenced MDA-MB-231 and CAL-62 cells show higher levels of ROS (Figure 12). In normoxia, MC3138 and lanthanum acetate alone or in combination increased ROS levels in both MDA-MB-231 and CAL-62. Hypoxia decreased the extent of ROS production. However, treatments with MC3138 and lanthanum acetate still increased ROS content (Figure 12). Mitochondria represent the most important source of ROS, and mitochondrial sirtuins are engaged for ROS control [47,48]. Our results show that: (i) mitochondrial ROS increased after GLS1 silencing and (ii) lanthanum acetate, MC3138 or lanthanum acetate plus MC3138 further increased mitochondrial ROS in normoxia and hypoxia.

## 5. Conclusions

In conclusion, our results show how the use of a selective activator of SIRT5 alone or in combination with a phosphate binder such as lanthanum acetate can represent a valid strategy to inhibit cell proliferation by reducing glutamine metabolism, mitophagy, and ultimately increasing total and mitochondrial ROS. While a therapy based on SIRT5 activation could be applied based on our previous in vivo [28] and present in vitro results, a strategy foreseeing a combination of SIRT5 activation and Pi binding could be considered only in the case of targeted and in situ therapy to avoid systemic effects [45].

## Data Availability

All data generated or analyzed during this study are included in this article.

## Supplementary Materials

The following supporting information can be downloaded at https://www.mdpi.com/article/10.3390/antiox12081635/s1. Figure S1: GAC expression in different human cell lines; Figure S2: Growth curve of wt and GLS1- MDA-MB-231 and CAL-62 cell lines; Figure S3: Global lysine acetylation after MC2791 and MC3138 treatment; Figure S4: Lanthanum acetate and MC3138 reduce colony formation and cancer cell growth of wt MDA-MB-231 cells; Figure S5: Lanthanum acetate and MC3138 reduce colony formation and cancer cell growth of MDA-MB-231 GLS1- cells; Figure S6: Gating strategy of wt and GLS1- MDA-MB-231 cells; Figure S7: Gating strategy of wt and GLS1- CAL-62 cells; Figure S8: Gating strategy of wt and GLS1- MDA-MB-231 cells under hypoxia; Figure S9: Gating strategy of wt and GLS1- CAL-62 cells under hypoxia; Figure S10: Autophagic flux in MDA-MB-231 cells; Figure S11: Autophagic flux in CAL-62 cells.
