# Supplementary material for: SIRT5 Activation and Inorganic Phosphate Binding Reduce Cancer Cell Vitality by Modulating Autophagy/Mitophagy and ROS
Source: Antioxidants (Basel). 2023 Aug 18;12(8):1635. doi: 10.3390/antiox12081635 (PMC10451763; doi:10.3390/antiox12081635)
Supplement: Supplementary file 1 [file antioxidants-12-01635-s001.zip › antioxidants-2449574-supplementary.pdf]

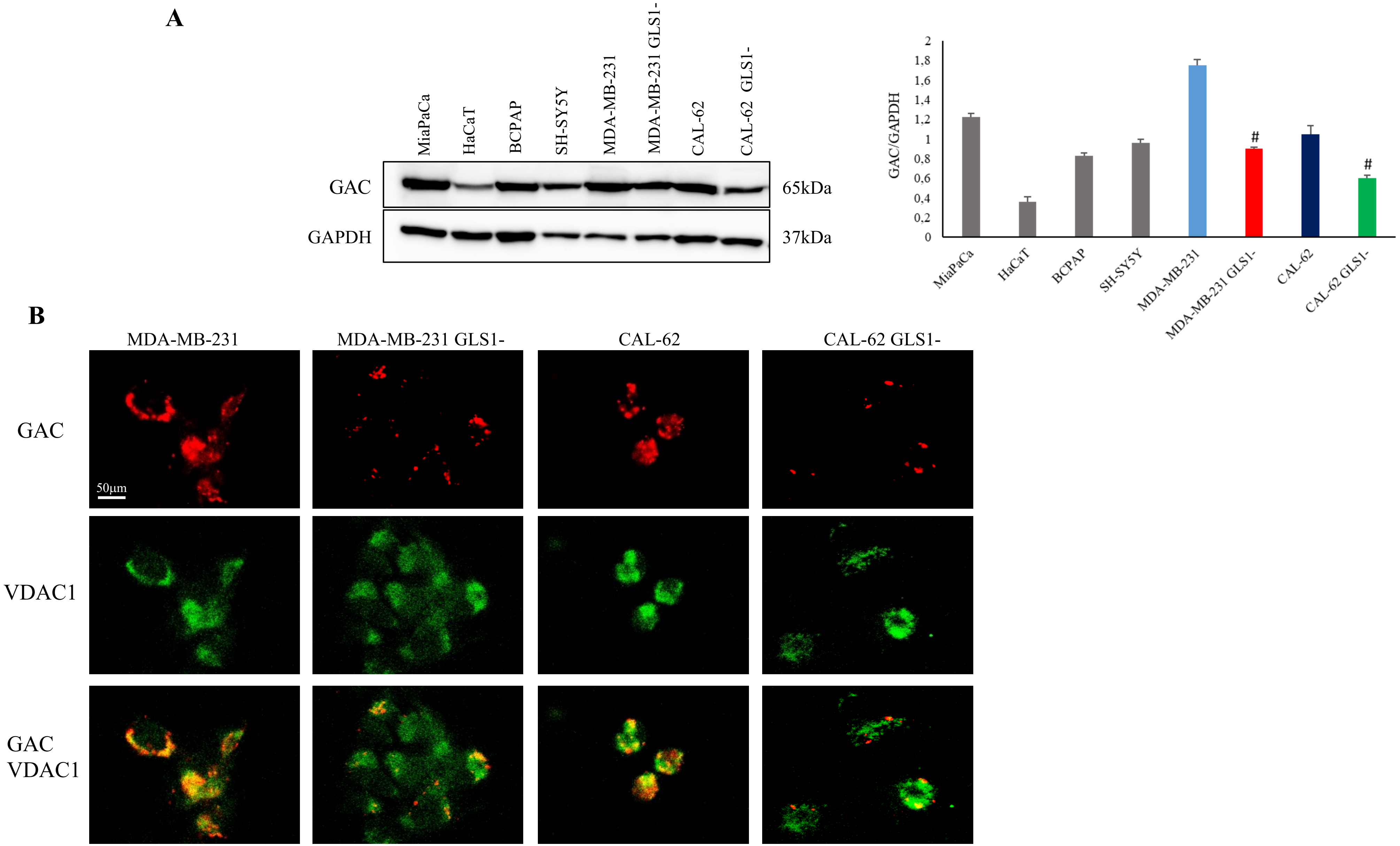

Figure S1. GAC expression in different human cell lines.

(A), Human cancer cell lines (MiaPaCa, BCPAP, SH-SY5Y, MDA-MB-231 and CAL-62) and keratinocytes cell line (HaCaT) were grown as described in Materials and Methods. MDA-MB-231 and CAL-62 cell lines were silenced for GLS1 as described in Materials and Methods. GAC expression was measured by Western blot. GAPDH was used as loading control. GAC expression was normalized with GAPDH and plotted as shown in the graph on the right side. # Significantly decreased compared with wt cells. #, $p < 0.05$.

(B), Confocal immunofluorescence analysis showing GAC and VDAC1 expression and mitochondrial localization in MDA-MB-231 and CAL-62 wt cells and GLS1- clones. Size bar is the same for each figure

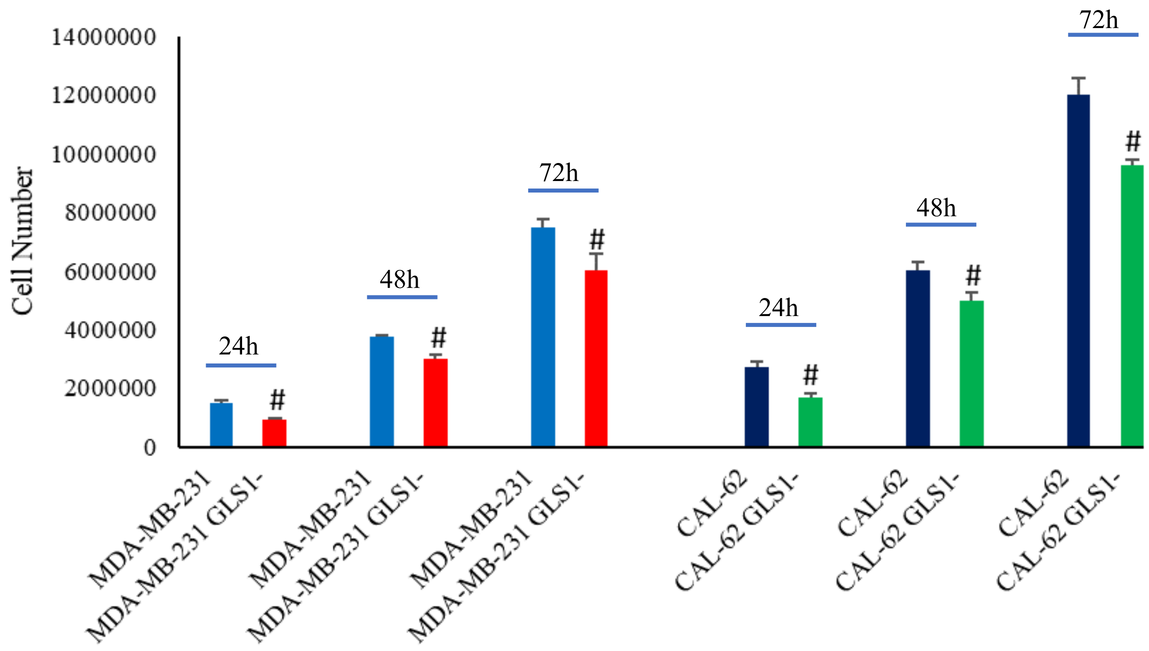

Figure S2. Growth curve of wt and GLS1- MDA-MB-231 and CAL-62 cell lines.
An equal number of wt and GLS1- MDA-MB-231 and CAL-62 cells was plated in 100mm dishes. Growth was assessed by counting living cells after 24, 48 and 72h as described in Materials and Methods. # Significantly decreased compared with the same time of wt cells. #, $p < 0.05$.

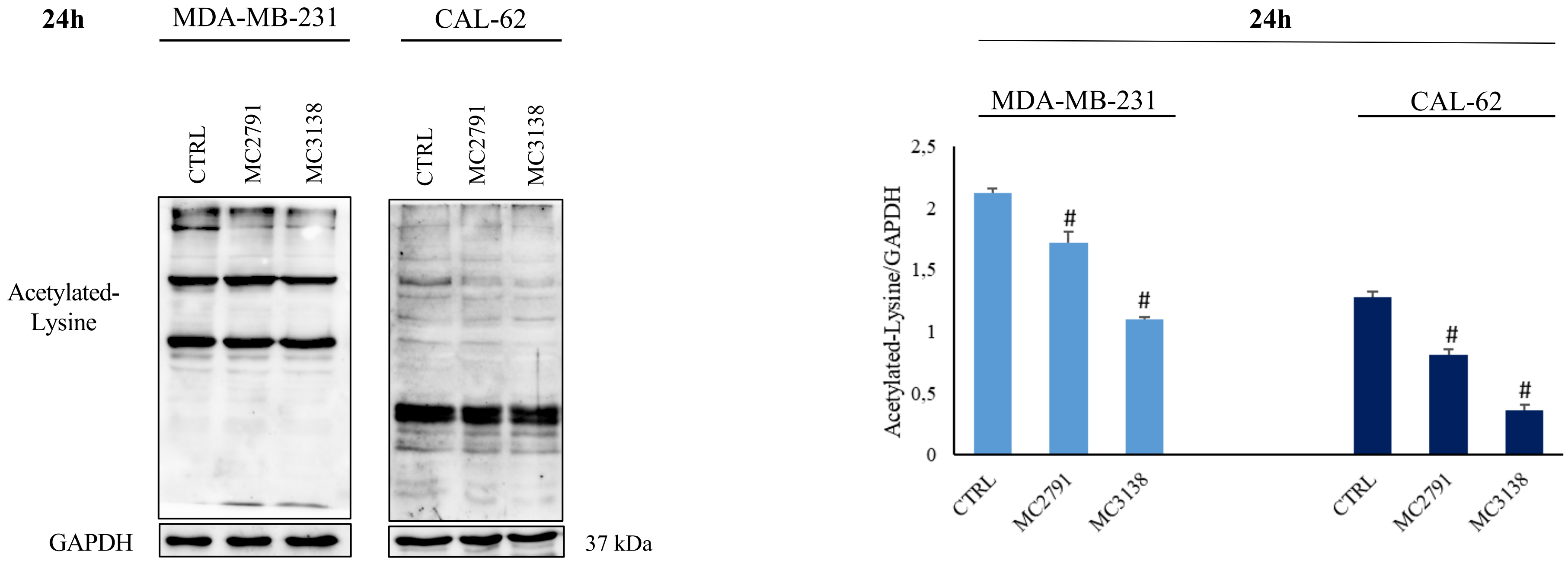

Figure S3. Global lysines acetylation after MC2791 and MC3138 treatment.
MDA-MB-231 and CAL-62 cells were either left untreated or treated with MC2791 (SIRT3 activator) or MC3138 (SIRT5 activator) for 24h. Acetylated lysines were determined as describe in Materials and Methods. GAPDH was used as loading control. Acetylated lysines expression was normalized with GAPDH and plotted as shown in the graph on the right side. # Significantly decreased compared with control cells. #, $p < 0.05$. CTRL, control.

MDA-MB-231 48h

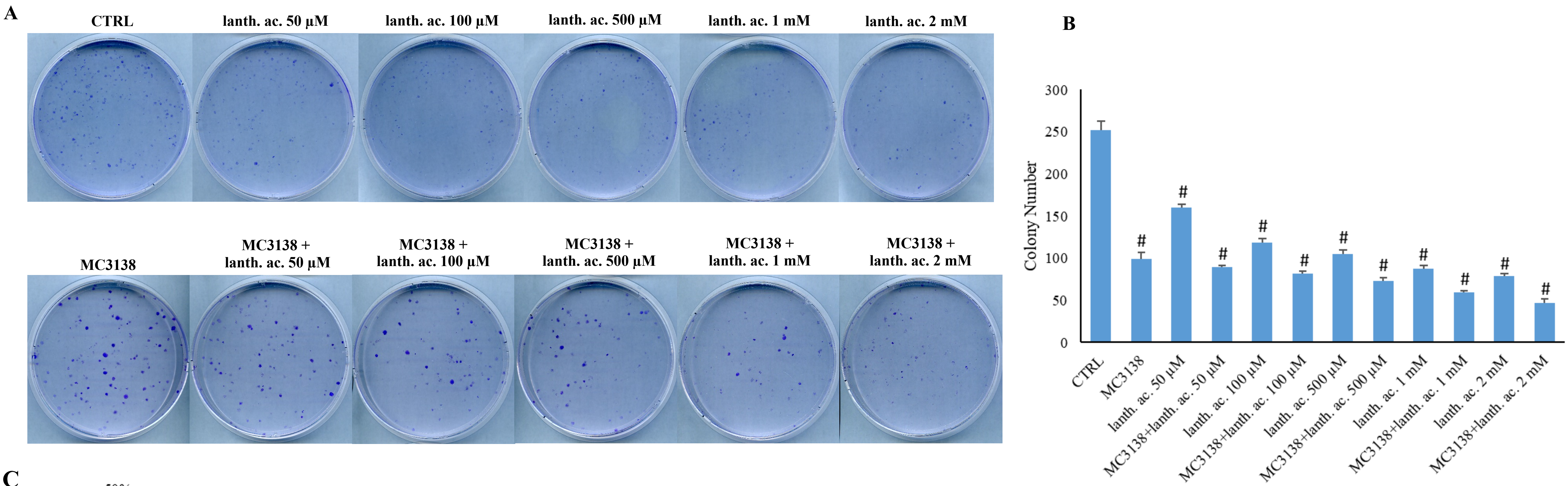

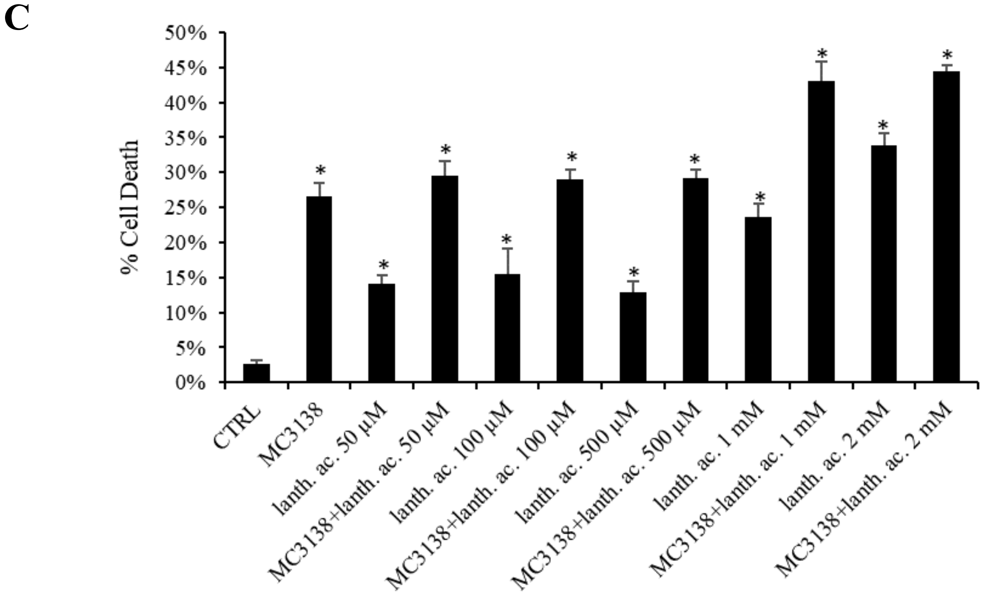

Figure S4. Lanthanum acetate and MC3138 reduce colony formation and cancer cell growth of wt MDA-MB-231 cells.
(A), MDA-MB-231 cells were either left untreated or treated for 48h with increasing concentrations of lanthanum acetate as indicated in the figure. Colony formation was obtained and determined as described in Materials and Methods. (B) Colonies were counted and plotted showing a decrease in number following the different treatments. (C) MDA-MB-231 cells were either left untreated or treated for 48h with increasing concentrations of lanthanum acetate as indicated. Cell death was determined by Trypan blue assay as described in Materials and Methods. * Significantly increased compared with untreated cells. *, $p < 0.05$. # Significantly decreased compared with control cells. #, $p < 0.05$. MC3138 was used at 50µM. CTRL, control; lanth. ac., lanthanum acetate.

MDA-MB-231 GLS1- 48h

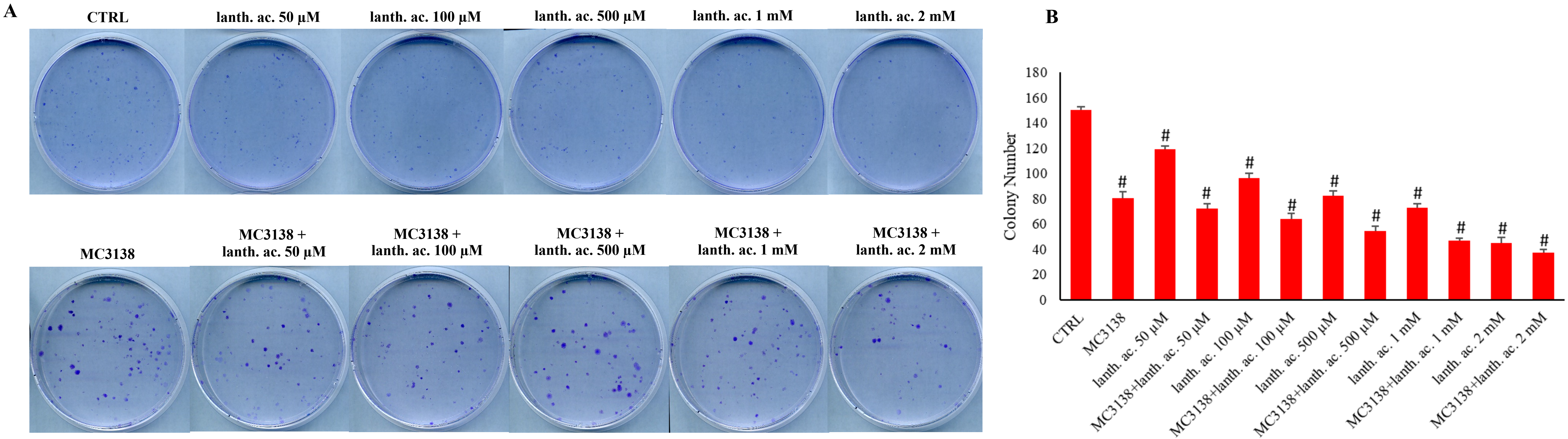

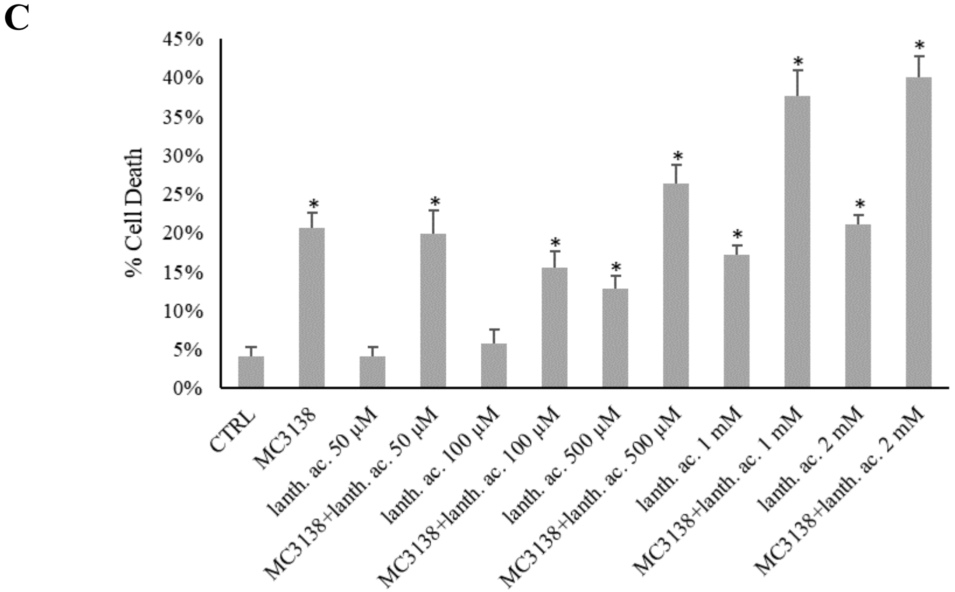

Figure S5. Lanthanum acetate and MC3138 reduce colony formation and cancer cell growth of MDA-MB-231 GLS1- cells.

(A), MDA-MB-231 GLS1- cells were either left untreated or treated for 48h with increasing concentrations of lanthanum acetate as indicated in the figure. Colony formation was obtained and determined as described in Materials and Methods. (B) Colonies were counted and plotted showing a decrease in number following the different treatments. (C) MDA-MB-231 GLS1- cells were either left untreated or treated for 48h with increasing concentrations of lanthanum acetate as indicated. Cell death was determined by Trypan blue assay as described in Materials and Methods. * Significantly increased compared with untreated cells. *, $p < 0.05$. # Significantly decreased compared with control cells. #, $p < 0.05$. MC3138 was used at 50µM. CTRL, control; lanth. ac., lanthanum acetate.

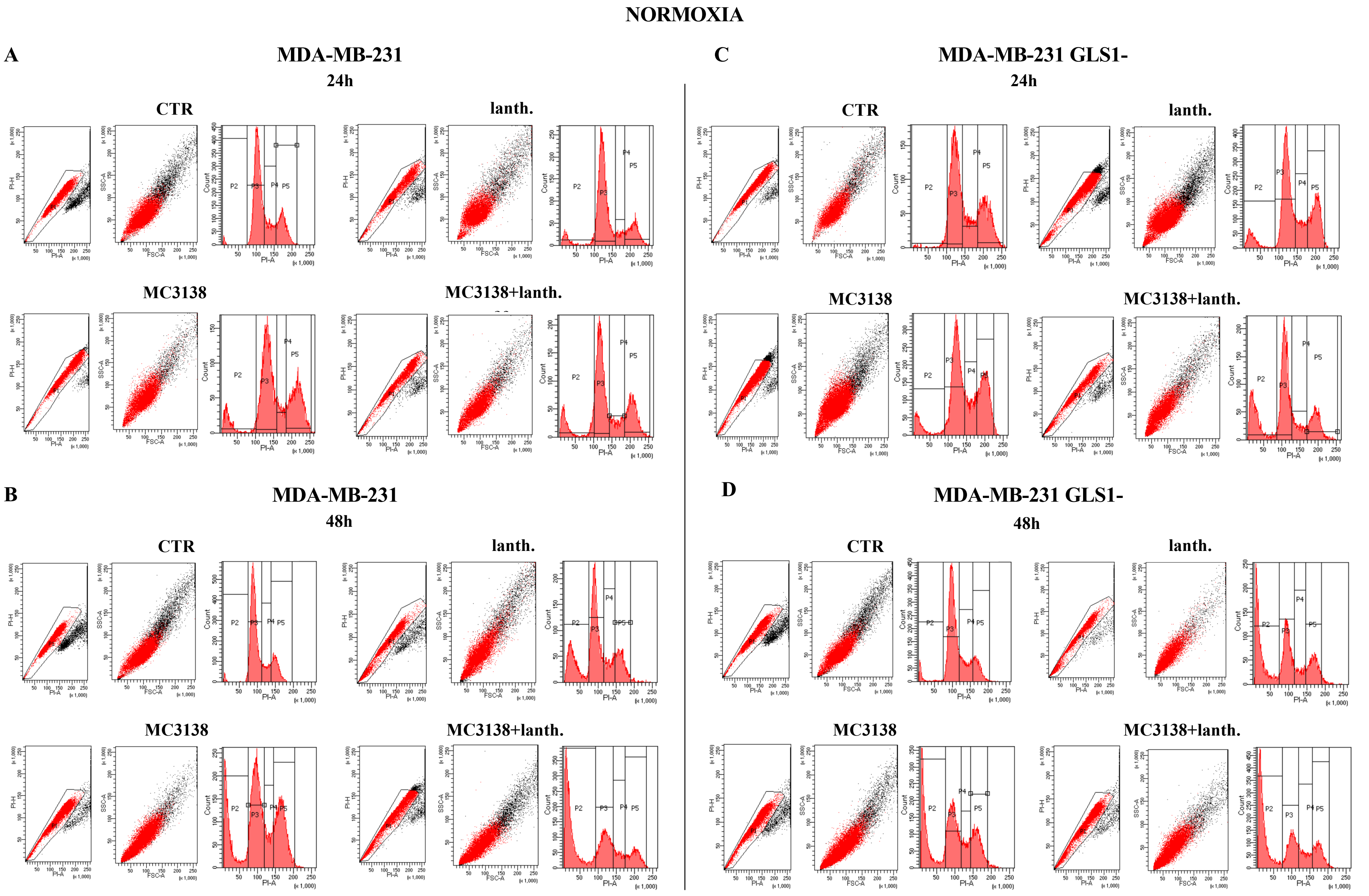

Figure S6. Gating strategy of wt and GLS1- MDA-MB-231 cells.
MDA-MB-231 wt and GLS1- cells were either left untreated or treated with lanthanum acetate, MC3138 or MC3138+lanthanum acetate for 24 and 48h. Representative gating strategy used to evaluate the cell cycle and the cell death is reported for each treatment.

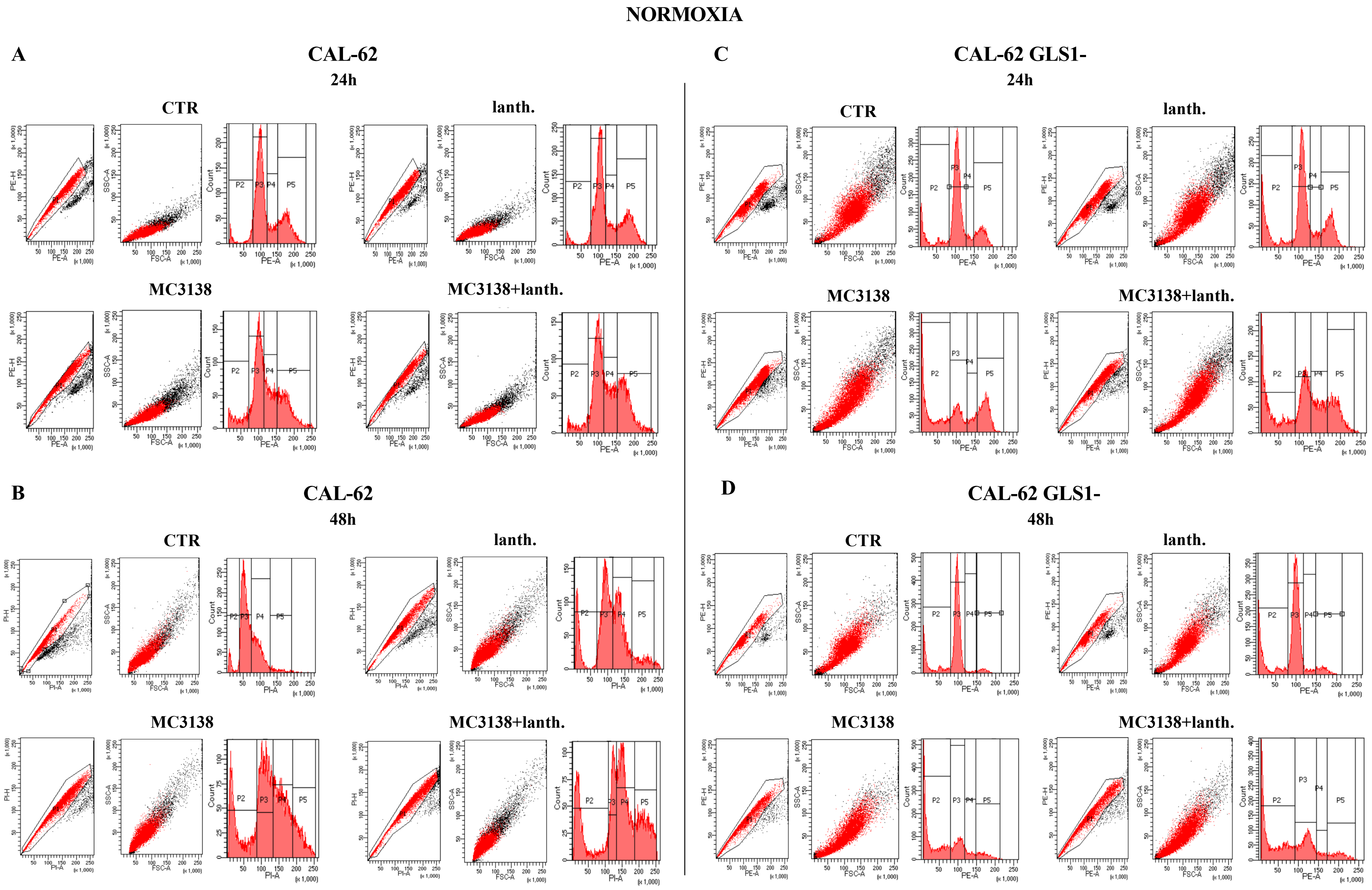

Figure S7. Gating strategy of wt and GLS1- CAL-62 cells.
CAL-62 wt and GLS1- cells were either left untreated or treated with lanthanum acetate, MC3138 or MC3138+lanthanum acetate for 24 and 48h. Representative gating strategy used to evaluate the cell cycle and the cell death is reported for each treatment.

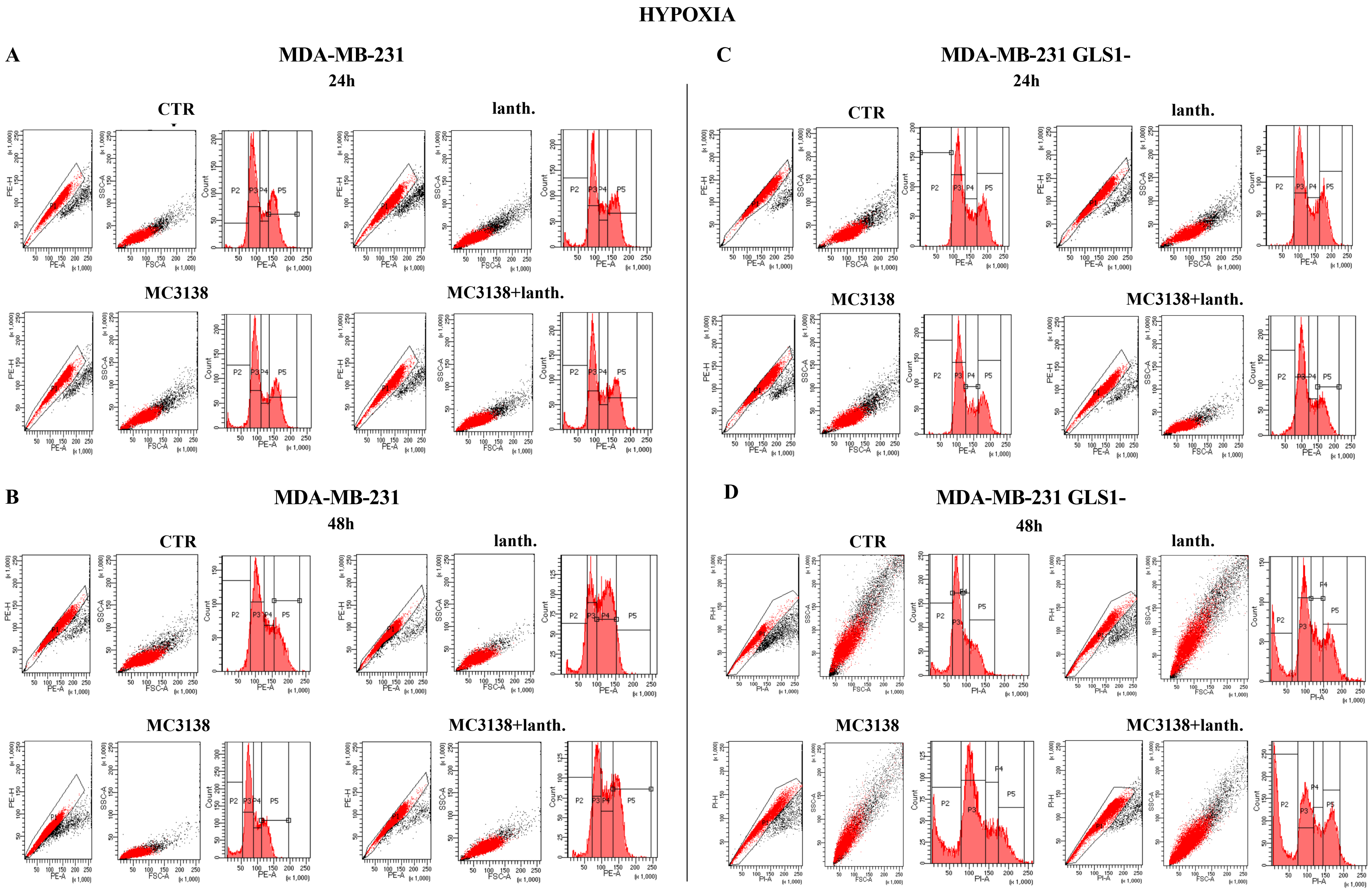

Figure S8. Gating strategy of wt and GLS1- MDA-MB-231 cells under hypoxia.
MDA-MB-231 wt and GLS1- cells were either left untreated or treated with lanthanum acetate, MC3138 or MC3138+lanthanum acetate for 24 and 48h in hypoxia. Representative gating strategy used to evaluate the cell cycle and the cell death is reported for each treatment.

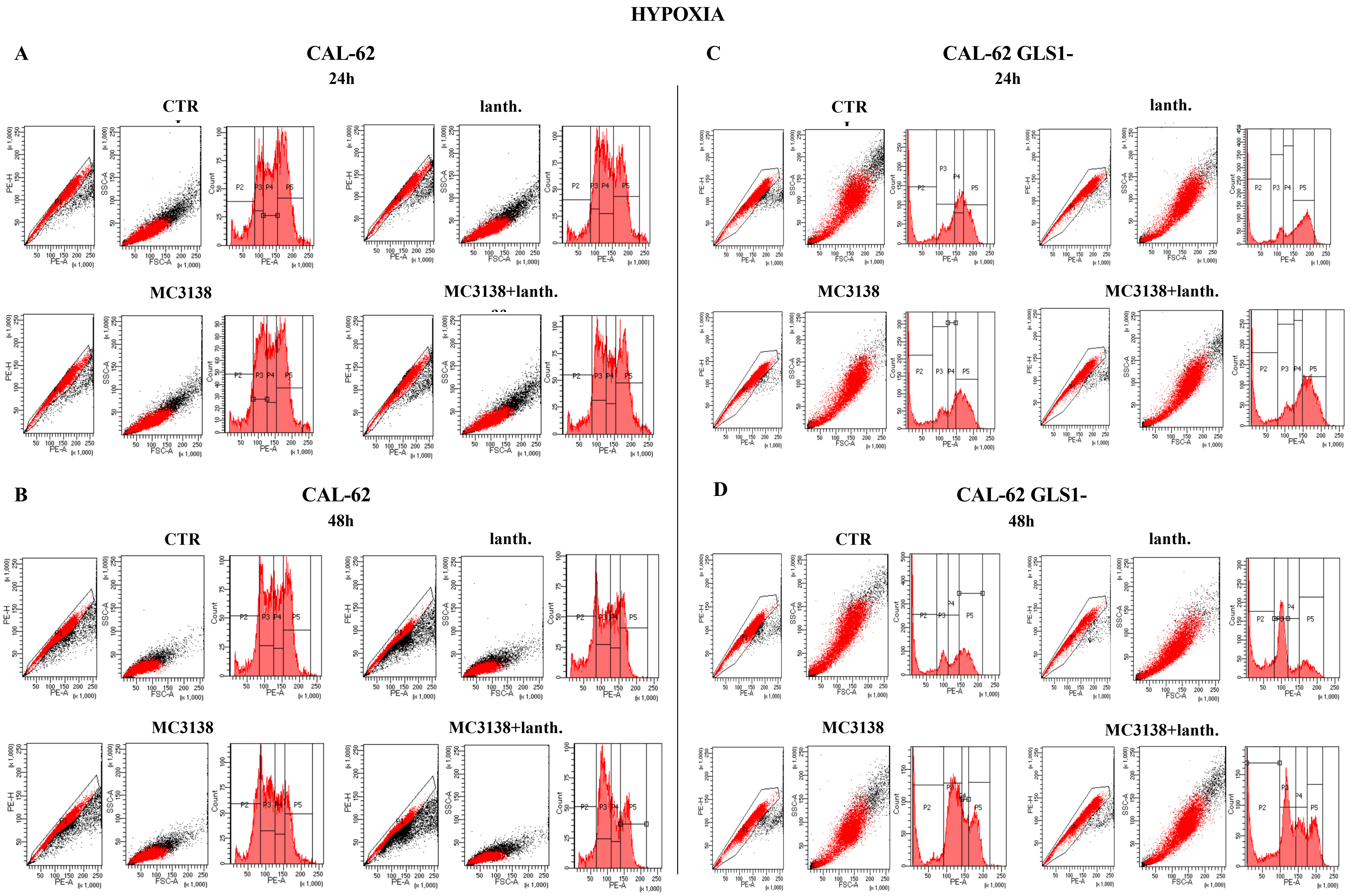

Figure S9 Gating strategy of wt and GLS1- CAL-62 cells under hypoxia.
CAL-62 wt and GLS1- cells were either left untreated or treated with lanthanum acetate, MC3138 or MC3138+lanthanum acetate for 24 and 48h in hypoxia. Representative gating strategy used to evaluate the cell cycle and the cell death is reported for each treatment.

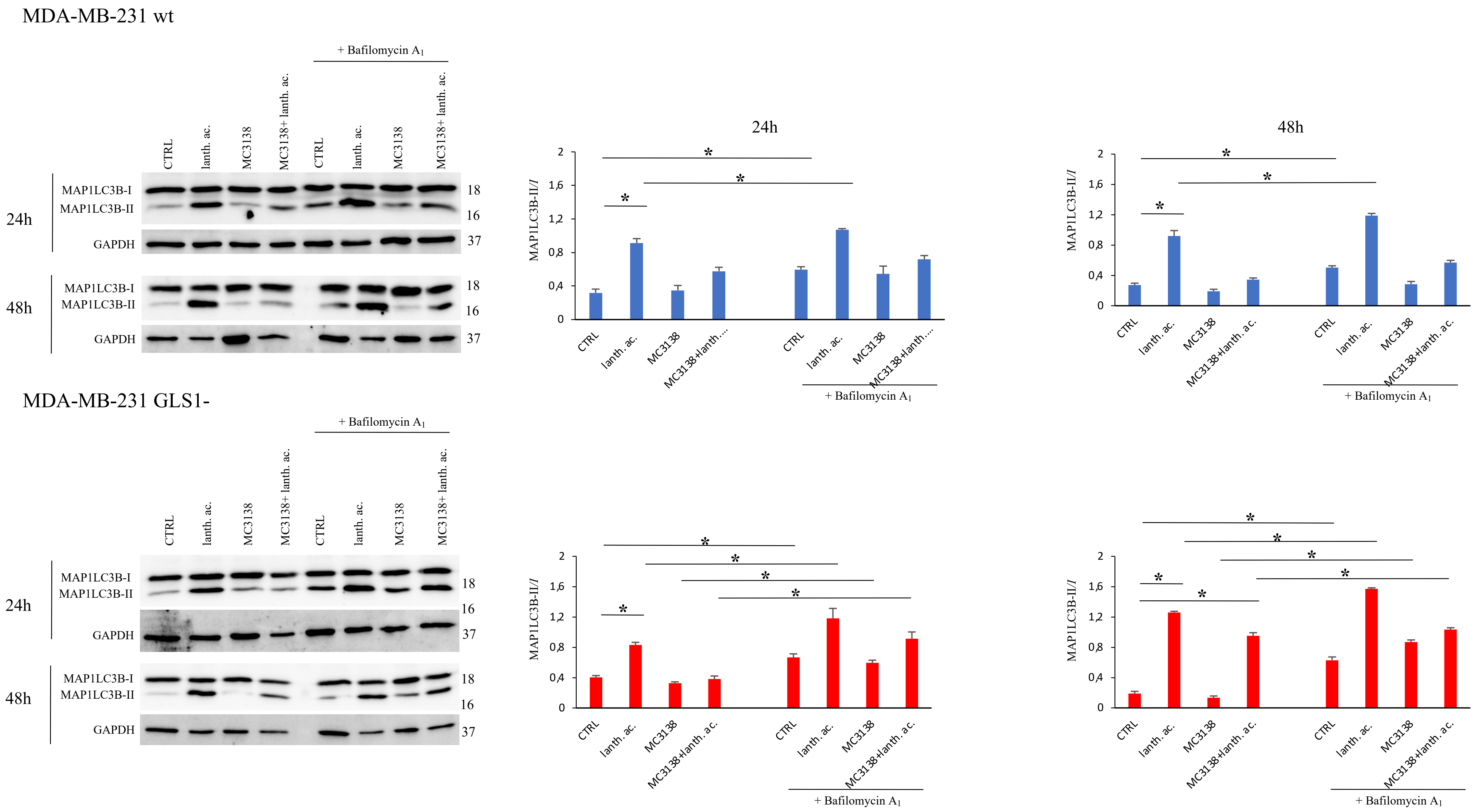

Figure S10. Autophagic flux in MDA-MB-231 cells.
MDA-MB-231 and MDA-MB-231 GLS1- cells were either left untreated or treated as indicated in the figure for 24 and 48h. Two hours before ending the treatments, 100 nM Bafilomycin A_1 was added to block autophagy. MAP1LC3B expression was determined by Western Blot as described in Materials and Methods. GAPDH was used as loading control. MAP1LC3B expression was normalized with GAPDH and plotted as shown in the graphs on the right side. Statistical significance between two treatments is indicated in the graphs by a bar and an asterisk. *, $p < 0.05$. CTRL, control; lanth ac., lanthanum acetate.

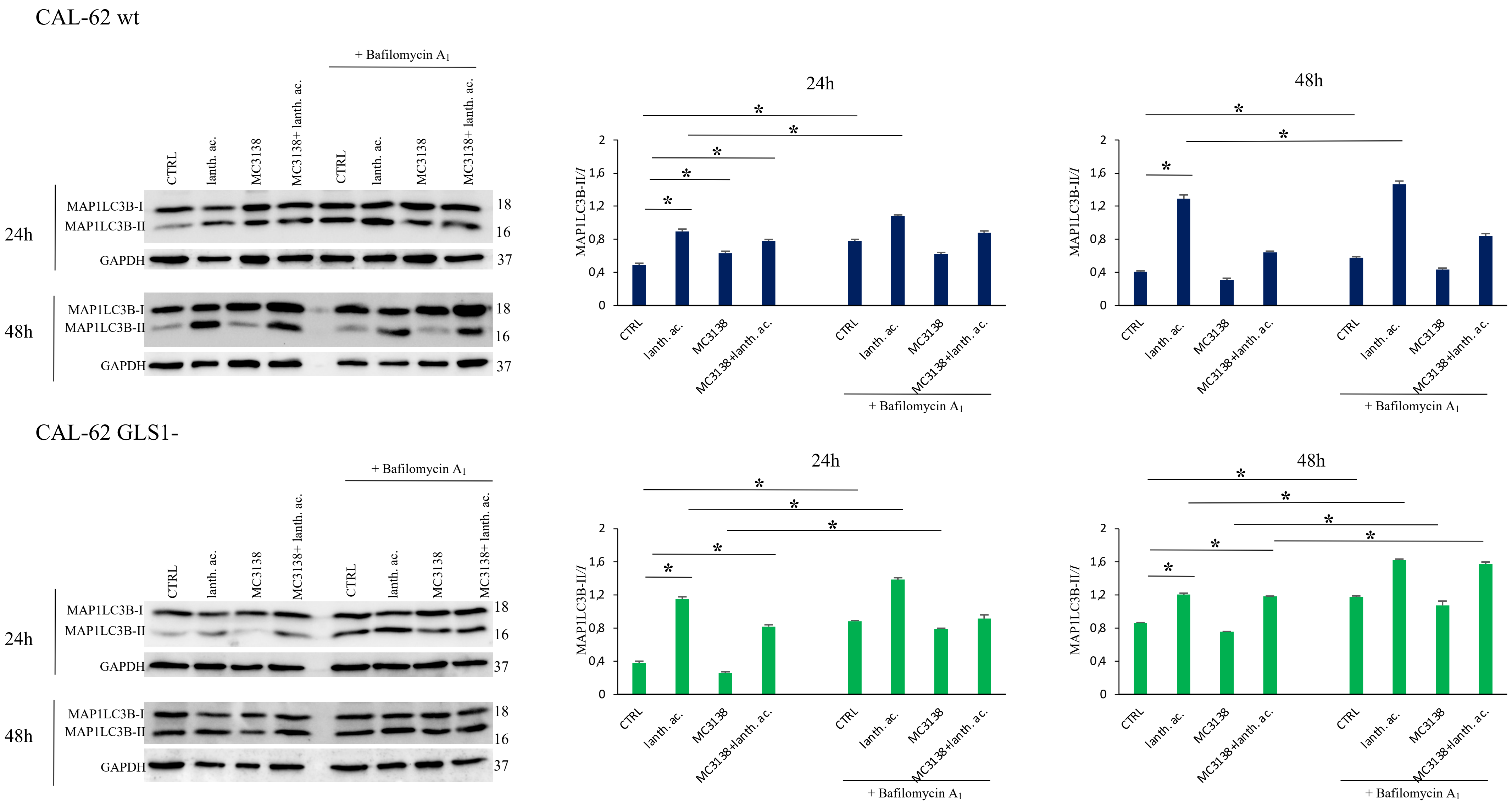

Figure S11. Autophagic flux in CAL-62 cells.
CAL-62 and CAL-62 GLS1- cells were either left untreated or treated as indicated for 24 and 48h. Two hours before ending the treatments, 100 nM Bafilomycin A_1 was added to block the autophagy. MAP1LC3B expression was determined by Western Blot as described in Materials and Methods. GAPDH was used as loading control. MAP1LC3B expression was normalized with GAPDH and plotted as shown in the graphs on the right side. Statistical significance between two treatments is indicated in the graphs by a bar and an asterisk. *, $p < 0.05$. CTRL, control; lanth ac, lanthanum acetate.
